# Molecular adaptations of the blood–brain barrier promote stress resilience vs. depression

**DOI:** 10.1073/pnas.1914655117

**Published:** 2020-01-23

**Authors:** Katarzyna A. Dudek, Laurence Dion-Albert, Manon Lebel, Katherine LeClair, Simon Labrecque, Ellen Tuck, Carmen Ferrer Perez, Sam A. Golden, Carol Tamminga, Gustavo Turecki, Naguib Mechawar, Scott J. Russo, Caroline Menard

**Affiliations:** ^a^Department of Psychiatry and Neuroscience, Université Laval, Quebec City, QC G1V 0A6, Canada;; ^b^CERVO Brain Research Center, Quebec, QC G1J 2G3, Canada;; ^c^Center for Affective Neuroscience, Nash Family Department of Neuroscience, Icahn School of Medicine at Mount Sinai, New York, NY 10029-5674;; ^d^Smurfit Institute of Genetics, Trinity College Dublin, Dublin 2, Ireland;; ^e^Department of Psychobiology, University of Valencia, 46010 Valencia, Spain;; ^f^Department of Biological Structure, University of Washington, Seattle, WA 98195;; ^g^Department of Psychiatry, University of Texas Southwestern Medical Center, Dallas, TX 75390;; ^h^Department of Psychiatry, McGill University, Montreal, QC H3A 1A1, Canada;; ^i^Douglas Hospital Research Centre, Montreal, QC H4H 1R3, Canada

**Keywords:** mood disorders, antidepressant, vascular, inflammation, epigenetic

## Abstract

Preclinical and clinical studies suggest that inflammation and vascular dysfunction contribute to the pathogenesis of major depressive disorder (MDD). Chronic social stress alters blood–brain barrier (BBB) integrity through loss of tight junction protein claudin-5 (cldn5) in male mice, promoting passage of circulating proinflammatory cytokines and depression-like behaviors. This effect is prominent within the nucleus accumbens, a brain region associated with mood regulation; however, the mechanisms involved are unclear. Moreover, compensatory responses leading to proper behavioral strategies and active resilience are unknown. Here we identify active molecular changes within the BBB associated with stress resilience that might serve a protective role for the neurovasculature. We also confirm the relevance of such changes to human depression and antidepressant treatment. We show that permissive epigenetic regulation of *cldn5* expression and low endothelium expression of repressive cldn5-related transcription factor *foxo1* are associated with stress resilience. Region- and endothelial cell-specific whole transcriptomic analyses revealed molecular signatures associated with stress vulnerability vs. resilience. We identified proinflammatory TNFα/NFκB signaling and *hdac1* as mediators of stress susceptibility. Pharmacological inhibition of stress-induced increase in hdac1 activity rescued *cldn5* expression in the NAc and promoted resilience. Importantly, we confirmed changes in *HDAC1* expression in the NAc of depressed patients without antidepressant treatment in line with CLDN5 loss. Conversely, many of these deleterious *CLDN5*-related molecular changes were reduced in postmortem NAc from antidepressant-treated subjects. These findings reinforce the importance of considering stress-induced neurovascular pathology in depression and provide therapeutic targets to treat this mood disorder and promote resilience.

## Significance

Thirty to fifty percent of depressed individuals are unresponsive to commonly prescribed antidepressant treatments, suggesting that biological mechanisms, such as stress-induced inflammation and blood vessel dysfunction, remain untreated. The blood–brain barrier is the ultimate frontier between the brain and harmful toxins or inflammatory signals circulating in the blood. Depression and vulnerability to chronic social stress are associated with loss of this barrier integrity; however, the mechanisms involved remain poorly understood. Identification of adaptations leading to resilience under stressful conditions could help develop novel treatments. Here we combined behavioral, pharmacological, and cell-specific gene profiling experiments in mice with epigenetic, molecular, and anatomical analysis of human samples to unravel mechanisms with therapeutic potential to protect the brain and promote resilience.

Major depressive disorder (MDD) will affect one out of five individuals throughout their lifetime and is now considered the leading cause of disabilities worldwide (1). Depression is a recurrent condition and only 30% of patients completely remit. This lack of efficacy suggests that traditional treatments do not address important causal biological factors (2). Clinical studies report higher prevalence of MDD in patients suffering from inflammatory conditions such as cardiovascular diseases or stroke, indicating that increased inflammation and vascular dysfunction could contribute to depression pathogenesis (2–5). Chronic stress is the primary environmental risk factor for depression and the nucleus accumbens (NAc) is a forebrain nucleus known to play a crucial role in stress responses (6). We recently showed that chronic social stress induces blood–brain barrier (BBB) leakiness in the nucleus accumbens (NAc) of male mice, promoting passage of circulating proinflammatory mediators and the establishment of depression-like behaviors, including social avoidance, anhedonia, and helplessness (7). Stress-induced increase in BBB permeability is mediated by loss of tight junction protein claudin-5 (cldn5), a major cell adhesion molecule which forms a paracellular barrier between endothelial cells (7–9). We showed that *CLDN5* expression is reduced in the NAc of depressed patients (7) in line with clinical studies reporting altered cerebrospinal fluid to serum ratio of peripheral markers in depression indicative of greater BBB permeability (10). Nevertheless, the mechanism underlying stress-induced reduction of cldn5 expression has yet to be determined. Moreover, some stressed mice are resilient in that they do not display depression-like behaviors and increased BBB permeability (7), suggesting that identification of active neurovascular adaptations within these resilient mice involved in maintenance of BBB integrity could represent an approach to develop innovative therapeutic strategies to treat mood disorders.

Here, we characterized molecular adaptations underlying stress vulnerability vs. resilience in the mouse NAc endothelial cells. Aberrant epigenetic modifications and transcriptional dysregulation have been associated with psychiatric disorders, including depression (11). Thus, we interrogated epigenetic changes induced by chronic social stress at the *cldn5* promoter in the NAc and found increased permissive acetylation of histones at the *cldn5* promoter within resilient mice. Next, we compared expression of Forkhead box protein O1 (*FoxO1*) and beta-catenin (*β-catenin*), two transcription factors known to inhibit cldn5 expression (12, 13) and found *FoxO1* to be reduced in resilient mice. We also performed NAc endothelial cell-specific transcriptomic analysis to reveal vascular genes and pathways involved in stress susceptibility vs. resilience. We identified histone deacetylase 1 (*hdac1*), an enzyme enriched in endothelial cells (14) and involved in transcriptional repression (15), as being up-regulated in stress-susceptible mice. Pharmacological inhibition of hdac1 activity in the NAc increased social interactions in stressed mice in line with rescue of cldn5 expression, providing a molecular framework for stress-induced alterations leading to BBB hyperpermeability and establishment of depression-like behaviors. Importantly, we confirmed increased *HDAC1* expression in the NAc of depressed patients and a significant correlation with *CLDN5* adding translational value to our mouse findings.

## Results

### Cldn5 Epigenetic Changes Are Associated with Stress Resilience vs. Depression.

**Fig. 1.**
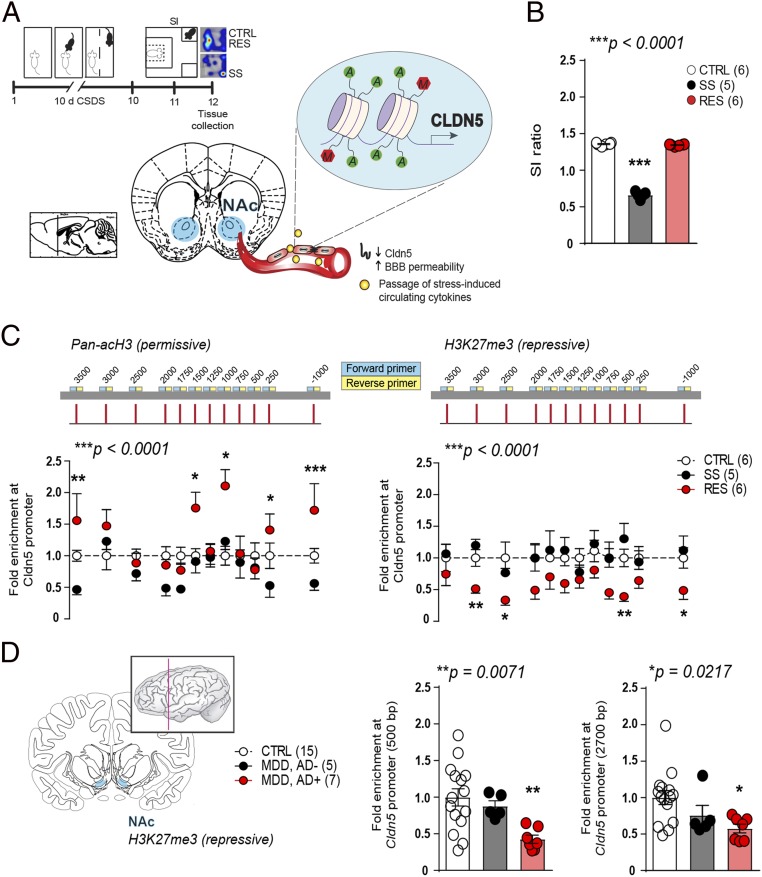
Permissive epigenetic regulation at the *cldn5* promoter is associated with stress resilience. (*A*) Experimental timeline and (*B*) behavioral profile of unstressed CTRL, SS, SI ratio <1, and RES, SI ratio >1 (one-way ANOVA: F_2,14_ = 1,084; ***P < 0.0001, *n* = 10 to 12 mice/group). (*C*) Stress resilience is associated with higher permissive pan-acetylation on histone 3 (pan-acH3, two-way ANOVA: phenotype effect F_2,168_ = 168; ***P < 0.0001) and lower repressive methylation on H3K27me3 (two-way ANOVA: phenotype effect F_2,168_ = 32.05; ***P < 0.0001) on *cldn5* gene promoter in the NAc of mice. Lower acetylation is also observed in SS mice when compared to CTRL (two-way ANOVA: phenotype effect F_1,108_ = 13.68; ***P = 0.0003). (*D*) Repressive histone methylation at *CLDN5* promoter is reduced in the NAc of major depressive disorder (MDD) subjects under antidepressant (AD^+^) treatment at time of death when compared to healthy CTRL or MDD subjects without treatment (MDD, AD^−^) (500 bp, one-way ANOVA: F_2,24_ = 6.119; **P = 0.0071; 2,700 bp, one-way ANOVA: F_2,24_ = 4.513; *P = 0.0217; *n* = 5 to 15 subjects/group). If one- or two-way ANOVA statistical test was significant, Bonferroni posttests were performed with *P < 0.05; **P < 0.01; ***P < 0.001. See *SI Appendix*, Tables S1 and S3 for primers and detailed demographic data.

Based on our findings that cldn5 expression is decreased in the NAc of depressed patients and of male mice following 10 d of chronic social defeat stress (CSDS) (7), we investigated whether an epigenetic mechanism is occurring at the *cldn5* promoter possibly leading to repression of its transcription and to vascular dysfunction. C57BL/6 mice were subjected to 10-d CSDS, a mouse model of depression, followed by a social interaction (SI) test 24 h later (Fig. 1*A*) to define stress-susceptible (SS) vs. resilient (RES) subpopulations according to their level of social interactions (16) when compared to unstressed controls (CTRL) (Fig. 1*B*, extended behavioral data *SI Appendix*, Fig. S1). NAc tissue was bilaterally punched, DNA fragmented and quantitative chromatin immunoprecipitation performed at permissive histone 3 acetylation (acH3) and repressive H3K27me3 sites (15). Primer pairs were designed to cover the promoter region up to ∼3,500 bp upstream of the transcription start site of *cldn5* (12) (*SI Appendix*, Fig. S2). Increased permissive acetylation and lower repressive methylation was observed only in RES mice, suggesting that stress-induced adaptive epigenetic changes are deficient in SS animals (Fig. 1*C*). Epigenetic modifications are also present at the *CLDN5* promoter in human depression, with lower repressive methylation in subjects with MDD treated by antidepressant medication at time of death (Fig. 1*D*). This is in line with our recent study showing that chronic treatment with the antidepressant imipramine can reverse social stress-induced loss of *cldn5* expression in stressed mice promoting resilience (7). These results suggest that chronic stress and depression affect cldn5 through changes in acetylation of histones that correlate with repressed transcription and subsequent impairment of BBB integrity. Conversely, compensatory changes are present in RES mice and MDD subjects treated with antidepressants possibly preventing loss of cldn5 and BBB dysfunction.

### Expression of Repressive cldn5-Related Transcription Factor FoxO1 Is Lower in Endothelial Cells of Resilient Mice.

**Fig. 2.**
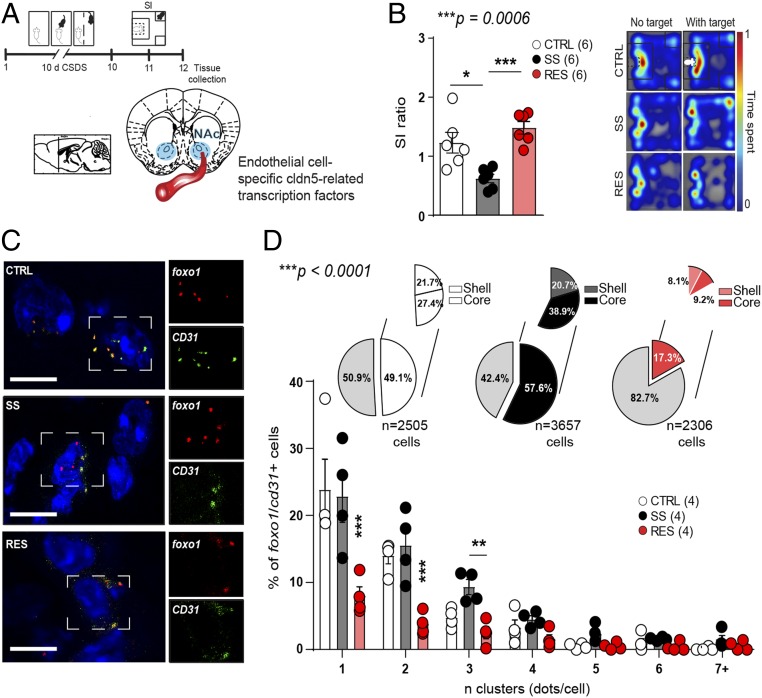
Expression of repressive cldn5-related transcription factor *FoxO1* is reduced in the endothelium of resilient mice. (*A*) Experimental timeline of a 10-d CSDS paradigm, SI test, and tissue collection for RNAscope in situ hybridization. (*B*) SI ratio of CTRL, SS, and RES mice (one-way ANOVA: F_2,15_ = 12.46; ***P = 0.0006, *n* = 6 mice/group). (*C*) Representative images of *FoxO1* expression in the NAc which is reduced in RES mice (two-way ANOVA: phenotype × *n* clusters interaction effect F_12,63_ = 4.856; ***P < 0.0001, *n* = 2,306 to 3,657 cells from four mice/group) (Scale bar, 10 μm) (*D*). Nonendothelial cells are identified in light gray in the pie charts and endothelial cells, double labeled with *CD31*, split between shell (white for CTRL, dark gray for SS, pink for RES) and core (white for CTRL, black for SS, red for RES) subregions of the NAc. If one- or two-way ANOVA statistical test was significant, Bonferroni posttests were performed with *P < 0.05; **P < 0.01; ***P < 0.001.

Vascular homeostasis is maintained by expression of endothelial tight junctions as well as adherens junctions formed by cldn5 and VE-cadherin, respectively (12). Adherens junctions are anchored to the actin cytoskeleton and influence organization and structure of tight junctions. In fact, both are involved in maintenance of BBB integrity and decreased VE-cadherin expression leads to *cldn5* transcriptional repression via FoxO1 and β-catenin (12, 17). Interestingly, FoxO1 was recently proposed as a novel gene predictor of depression in gene × environment interactions (18). On the other hand, β-catenin mediates stress resilience through modulation of microRNAs in NAc medium spiny neurons (19). Thus, we compared *FoxO1* and β-catenin gene *ctnnb1* expression in NAc endothelial cells following 10-d CSDS to evaluate if this signaling pathway is involved in stress-induced loss of BBB integrity (Fig. 2 *A* and *B*). Indeed, endothelial cell-specific *cdh5* (VE-cadherin gene) is reduced in the NAc of both SS and RES mice after CSDS (*SI Appendix*, Fig. S3 *A*–*C*), suggesting that molecular adaptations defining stress susceptibility vs. resilience may be present downstream of adherens junctions. Accordingly, *FoxO1* expression is increased in RES mice when compared to unstressed CTRL and SS animals; however, *FoxO1* is not specific to endothelial cells (*SI Appendix*, Fig. S3*D*). Thus, we took advantage of RNAscope in situ hybridization and quantified expression of cldn5-related transcription factors in endothelial cells only via double labeling with the endothelial cell-specific marker CD31 (Fig. 2 *C* and *D* and *SI Appendix*, Fig. S4 for representative full-length vessels). Following CSDS, mouse subpopulations were defined (Fig. 2*B*, extended behavioral data *SI Appendix*, Fig. S5), then brains were collected, sliced, stained, imaged, and semiautomated quantification was performed with MATLAB software. We observed a significant reduction of *FoxO1* expression in NAc endothelial cells of RES mice (Fig. 2 *C* and *D*). Conversely, no difference was noted for *ctnnb1* expression in NAc heterogenous preparation (*SI Appendix*, Fig. S3*D*) or endothelial cells (*SI Appendix*, Fig. S6). The NAc can be divided in two areas: a core and a shell subdivision with differences observed not only at the molecular level but also function for reward encoding and motivation (20). Changes in cldn5-related transcription factors were more present in the NAc shell vs. core of RES mice (*SI Appendix*, Fig. S7), reinforcing the presence of molecular changes associated with coping in this subpopulation of mice.

### Transcriptome-Wide Changes in NAc Endothelial Cells Are Involved in Stress Susceptibility.

**Fig. 3.**
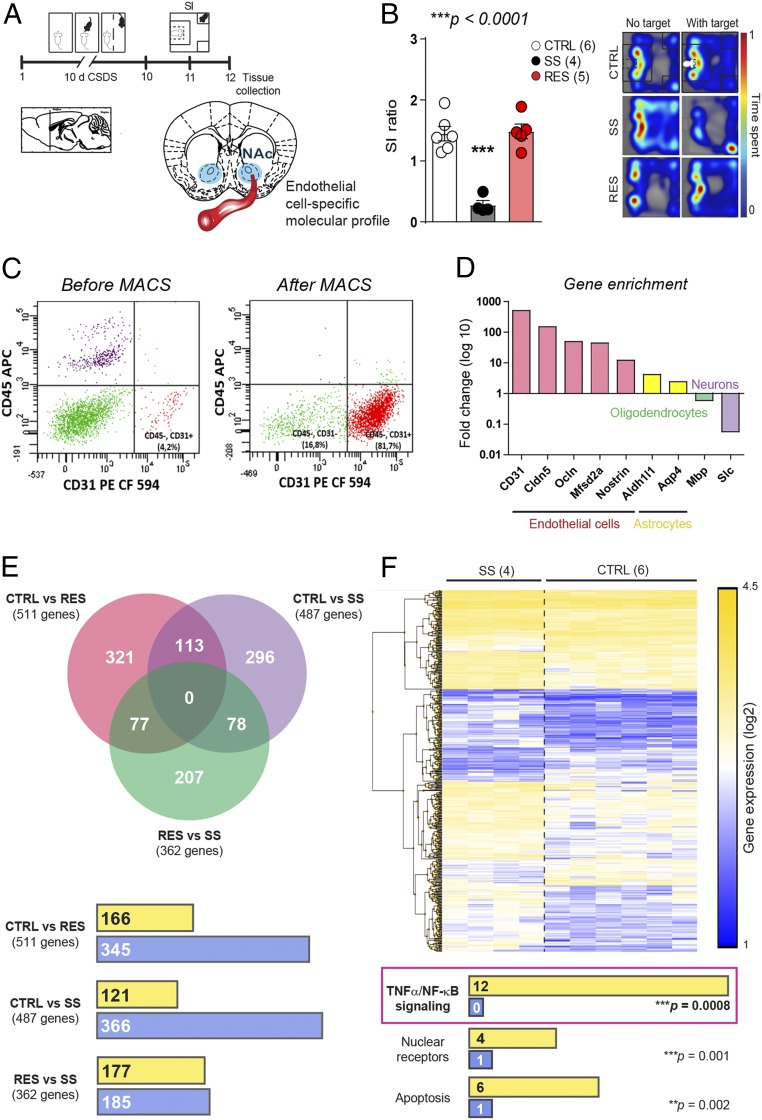
Stress-induced transcriptome-wide changes in NAc endothelial cell gene expression. (*A*) Experimental timeline and (*B*) behavior profiles of CTRL, SS, and RES mice used to compare NAc endothelial cell gene expression (one-way ANOVA: F_2,12_ = 31.44; ***P < 0.0001, *n* = 4 to 6 mice/group). (*C*) Enrichment of NAc endothelial cells following MACS was confirmed by flow cytometry (*C*, 81.7% vs. 4.2% for heterogenous preparation including all cell types) and (*D*) quantitative PCR (500- to 12-fold enrichment for endothelial cell-related genes vs. 4- to 2-fold for astrocytes, 0.6-fold for oligodendrocytes, and 0.06-fold for neurons after MACS purification vs. heterogenous preparation). (*E*) Venn diagrams revealed poor overlap of gene expression changes when group comparisons were performed. Gene expression was mostly down-regulated (blue) in NAc endothelial cells of stressed mice when compared to unstressed controls with a similar number of genes being up-regulated (yellow) vs. down-regulated (blue) in RES vs. SS animals. Significance was set at ±2-fold change and *P* < 0.05. See Dataset S1 for detailed gene lists. (*F*) Hierarchical clustering heat map of SS vs. CTRL mice and biological pathways including the higher number of genes up-regulated (yellow) or down-regulated (blue) in the NAc endothelial cells of these groups of mice. One-way ANOVA statistical test was significant thus Bonferroni posttests were performed with ***P < 0.001.

To identify region- and cell-specific molecular alterations leading to stress-induced loss of cldn5, BBB permeability, and depression-like behaviors, we performed transcriptome-wide gene-level expression profiling in the NAc of CTRL, SS, and RES mice following magnetic-activated cell sorting (MACS) of endothelial cells. This technique exploits immunomagnetic microbeads to quickly and gently separate cell types, preventing cell activation which is a confounding factor with other techniques such as fluorescent activated cell sorting (FACS). Mice were subjected to 10 d of CSDS, behavioral phenotype was defined, and NAc punches were collected 24 h later and immediately processed through MACS purification (Fig. 3 *A* and *B*, extended behavioral data *SI Appendix*, Fig. S8). Enrichment of endothelial cells was confirmed by FACS (Fig. 3*C*) and increased expression of genes specific to endothelial cells (*CD31*, *cldn5*, *ocln*, *Mfsd2a*, *nostrin*) was observed compared to those expressed in astrocytes (*aldh1l1*, *aqp4*), oligodendrocytes (*mbp*), or neurons (*slc17a6*) (Fig. 3*D*). RNA was extracted and transcriptome profiling done with the mouse Clariom S assay, which allows measurement of gene expression from >20,000 well-annotated genes. Transcriptomic profiling revealed low overlap of changes between each mouse group confirming that different stress-induced adaptations occur in SS vs. RES mice, promoting BBB leakiness vs. maintenance of BBB integrity, respectively (Fig. 3*E* and Dataset S1 for gene lists for each group comparison). Analysis of biological pathways differentially regulated between NAc endothelial cells of SS and CTRL mice revealed increased expression of genes associated with the proinflammatory tumor necrosis factor alpha/nuclear factor kappa-light-chain-enhanced of activated B cells (TNFα/NFκB) pathway (Fig. 3*F* and *SI Appendix*, Fig. S9 for scatter and volcano plots with identified genes). Up-regulation of TNFα/NFκB signaling decreases tight junction protein expression leading to increased BBB permeability in pathological conditions such as stroke (21) and here we report that this pathway is also involved in stress-induced BBB leakiness in the mouse NAc.

### Inhibition of Stress-Induced Increased *hdac1* Expression Rescues *cldn5* Expression Promoting Resilience.

**Fig. 4.**
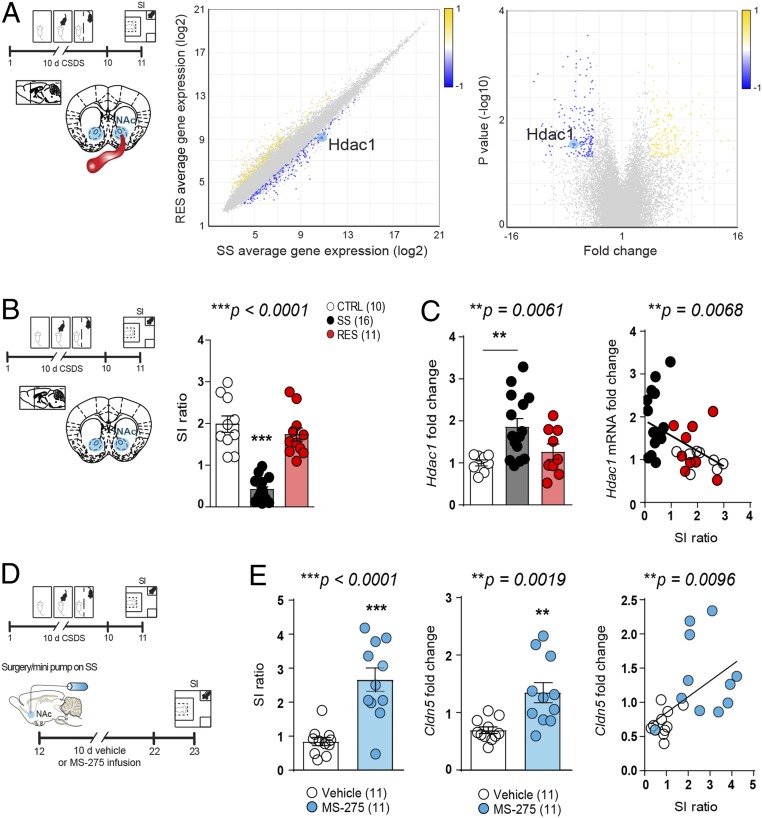
Inhibition of stress-induced increased *hdac1* expression promotes resilience and rescues *cldn5* expression. (*A*) Following 10 d of CSDS, *hdac1* expression is down-regulated in the NAc endothelial cells of RES mice when compared to SS mice (one-way ANOVA, *P = 0.0301, *n* = 4 to 5 mice/group). (*B* and *C*) Higher stress-induced *hdac1* level in the NAc of SS mice was confirmed by quantitative PCR and negatively correlated with SI (SI ratio: one-way ANOVA: F_2,34_ = 44.27; ***P < 0.0001; *hdac1* fold change: one-way ANOVA: F_2,29_ = 6.122; **P = 0.0061, Pearson’s correlation between SI ratio and *hdac1* fold change: **P = 0.0068, *n* = 10 to 16 mice/group). (*D*) Mice were screened with the SI test following 10 d of CSDS and split equally in two groups then administered vehicle or MS-275. (*E*) A second SI test was performed after 10 d of treatment revealing increased social interactions in the MS-275–treated group (unpaired *t* test: ***P < 0.0001) in line with higher *cldn5* expression (Pearson’s correlation: **P = 0.0019, *n* = 11 mice/group). One-way ANOVAs statistical test were significant, thus Bonferroni posttests were performed with **P < 0.01; ***P < 0.001.

We next explored transcripts differentially regulated in the NAc endothelial cells of SS vs. RES mice to identify genes involved in BBB molecular adaptations underlying stress susceptibility vs. resilience. We found a 3-fold reduction in expression of histone deacetylase 1 (*hdac1*), an enzyme known to play a key role in the regulation of gene expression, in NAc endothelial cells of RES mice when compared to SS mice (Fig. 4*A*), suggesting that *hdac1* may prevent coping-related molecular adaptations. Interestingly, *hdac1* seems to be most highly expressed in endothelial cells compared to other cell types (*SI Appendix*, Fig. S10). Increased *hdac1* expression in the NAc of SS mice was confirmed by quantitative qPCR and significantly correlated with the level of social interactions (Fig. 4 *B* and *C*). To assess whether increased *hdac1* expression plays a causal role in stress-induced cldn5 loss, we used a pharmacological approach by inhibiting class I HDAC (including both Hdac1 and Hdac3) activity directly into the NAc with MS-275, as this compound, like other HDAC inhibitors, has low brain uptake when administered i.v. (22). Mice were subjected to 10 d of CSDS, then SS mice were treated with either vehicle or MS-275 for 10 d (15) (Fig. 4*D*). Administration of MS-275 increased social interactions in line with a rescue of *cldn5* expression in the NAc of treated SS mice (Fig. 4*E*). Changes in *hdac* expression and activity have previously been reported in CSDS (15) and human depression (23); here we show that these changes are associated with stress-induced BBB leakiness and vascular molecular signaling.

### Human Depression Is Associated with Molecular Changes in the NAc Endothelium.

**Fig. 5.**
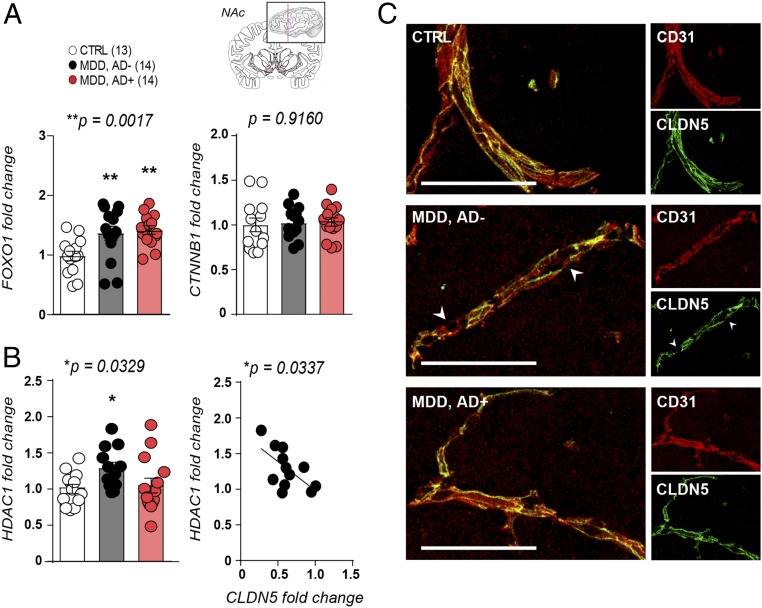
Increased *HDAC1* expression is associated with CLDN5 loss in human depression. (*A*) *FOXO1* expression is increased in the NAc of MDD patients with or without antidepressant treatment when compared to healthy controls (one-way ANOVA: F_2,38_ = 7.616; **P = 0.0017, 13 to 14 subjects/group). No change was observed for *CTNNB1* (one-way ANOVA: F_2,39_ = 0.08791; *P* = 0.9160, *n* = 13 to 14 subjects/group). (*B*) *HDAC1* expression is increased in the NAc of MDD patients without antidepressant treatment (one-way ANOVA: F_2,36_ = 3.865; *P = 0.0329) and correlated with *CLDN5* mRNA level (Pearson’s correlation: *P = 0.0337, *n* = 12 to 14 subjects/group). (*C*) CLDN5 protein expression is reduced in postmortem NAc samples of depressed subjects when compared to healthy controls. (Scale bar, 100 μm.) See *SI Appendix*, Tables S1 and S3 for primers and detailed demographic data.

To confirm translational value of our mouse findings we evaluated cldn5-related transcription factors and *HDAC1* expression in NAc postmortem samples from healthy controls vs. MDD patients with or without antidepressant treatment at time of death. In line with our mouse data, *FOXO1* expression was altered in the NAc of depressed patients, independently of treatment, while no change was observed for *CTNNB1* (Fig. 5*A*). Conversely, *HDAC1* level was only increased in the NAc of MDD patients without antidepressant treatment (Fig. 5*B*). Moreover, increased *HDAC1* expression was significantly correlated with lower *CLDN5* levels in the NAc of untreated depressed patients, in line with a loss of CLDN5 proteins in blood vessels (Fig. 5*C*), reinforcing the link between this epigenetic regulator and cldn5-mediated loss of BBB integrity.

## Discussion

**Fig. 6.**
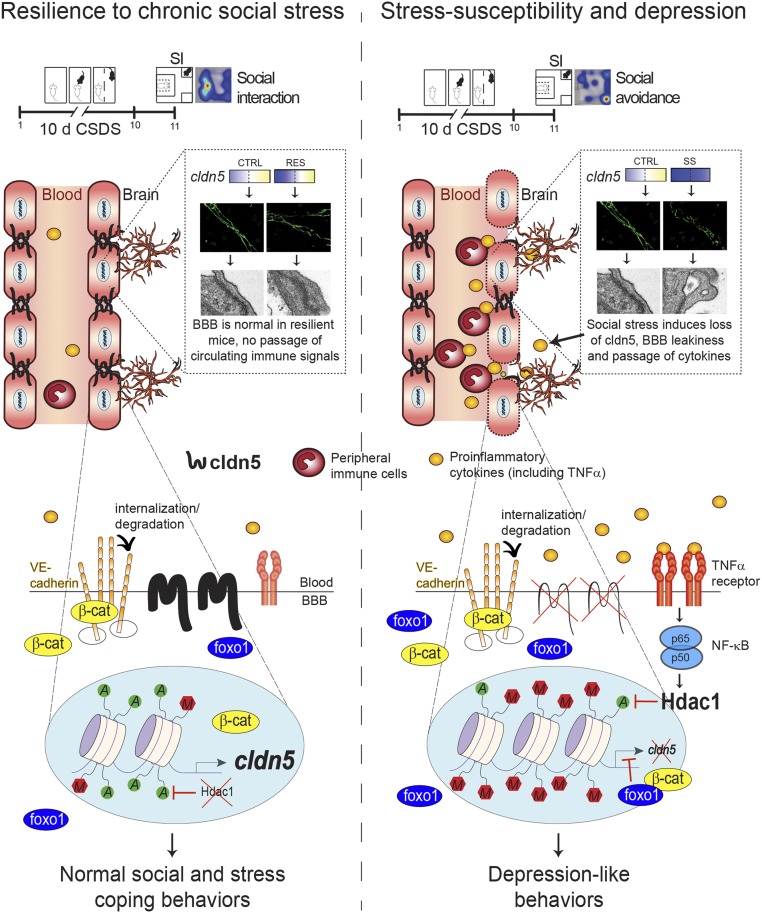
Claudin5 (cldn5) permissive epigenetic regulation is associated with stress resilience while lack of endothelial molecular adaptations and inflammation leads to cldn5 loss and depression. Low *hdac1* expression and high acetylation/low methylation on the *cldn5* gene promoter allows maintenance of the BBB integrity under chronic stress leading to normal social and stress coping behaviors. These proresilient adaptive changes also include reduced expression of the repressive transcription factor *Foxo1*, possibly to prevent its binding to the *cldn5* promoter now accessible through permissive epigenetic changes, and subsequent suppression of *cldn5* production (*Left*). Conversely, endothelial inflammation mediated by high levels of circulating proinflammatory cytokines (7, 33), activation of TNFα/NFκB signaling, and elevated *hdac1* activity prevents proper molecular adaptations, including access to the *cldn5* gene promoter, leading to cldn5 loss. Stress-induced loss of this tight junction protein results in BBB hyperpermeability, passage of circulating proinflammatory mediators, and the establishment of depression-like behaviors (*Right*). Adapted with permission from ref. 7.

We describe here a molecular framework in which cldn5-related epigenetic modifications and transcriptional changes in the NAc lead to stress resilience or vulnerability and depression. In rodents, depression-like behaviors have been associated with increased BBB permeability (7, 8) while vascular dysfunction was not observed in those displaying stress resiliency (7, 24). However, the cellular and molecular mechanisms involved in this process remained elusive. Here, we report that normal social interactions are related to permissive epigenetic and transcriptional regulation of the *cldn5* gene promoter allowing maintenance of BBB integrity despite stressful conditions (Fig. 6). Conversely, stress-induced BBB permeability is linked to inflammation of the endothelium and up-regulation of epigenetic repressor hdac1, which reduces cldn5 expression and may lead to loosening of tight junctions, BBB leakiness, and establishment of depression-like behaviors.

Only a handful of studies have explored molecular and epigenetic regulation of cldn5 despite its role in several inflammatory conditions and neurological disorders, including stroke (25), glioblastoma (26), multiple sclerosis, and Alzheimer’s disease (27). In an elegant set of papers, Taddei et al. showed that VE-cadherin mediates up-regulation of cldn5 and epigenetic regulation of endothelial gene expression (12, 13). We observed that VE-cadherin is down-regulated in both SS and RES mice following chronic social stress, suggesting that cellular and molecular changes determining behavioral phenotype are occurring downstream of endothelial adherens junctions. Cldn5-related repressive transcription factor *Foxo1* expression is reduced in the NAc endothelial cells of RES mice which could represent a compensatory change to prevent cldn5 loss. Interestingly, *FOXO1* was recently identified as a gene predictor of depression in gene × environment interactions taking into account early life traumatic experiences (18). In our human cohort, we observed an increase of *FOXO1* expression in the NAc of depressed patients, reinforcing possible involvement of this transcription factor in mood disorders. FOXO1 regulates vascular endothelial growth factor A (VEGFA) expression and promotes angiogenesis in healing wounds (28). Under inflammatory conditions, VEGF increases vascular permeability (29), an effect mediated by cldn5 disruption (30). It will be interesting to further evaluate endothelial cell signaling in prospective studies. However, assessment of posttranslational modifications in a cell- and region-specific manner in the mouse brain is challenging due to the limited amount of tissue.

Our transcriptional analysis of the mouse endothelium revealed increased expression of inflammatory mediators in the NAc of SS mice with a prominent role for the TNFα/NFκB signaling pathway. Neuroimmune mechanisms of depression have been receiving increasing attention in the past years (3, 31, 32) and therapeutic approaches aimed at reducing inflammation are currently ongoing in a handful of clinicals trials (2). Indeed, reducing circulating proinflammatory cytokines has a proresilient effect in stressed mice (33) and elevated baseline inflammation, including heightened blood TNFα level, has been reported in MDD patients (2, 3, 34). TNFα-induced NFκB signaling represses cldn5 expression in mouse brain endothelial cells (35), suggesting that reducing circulating TNFα may prevent social stress-induced loss of BBB integrity in the NAc. Interestingly, Cheng et al. reported that i.p. treatment with the TNFα inhibitor Etanercept reduces hippocampal BBB permeability induced by learned helplessness (8). NFκB signaling has been linked to impaired neurogenesis in the hippocampus (36) and immature synapse formation in the NAc (37) promoting depressive behaviors. Our results demonstrate that endothelium TNFα/NFκB signaling is also a key regulator of social stress responses. The BBB has been a major challenge to overcome in the development of novel efficient antidepressant drugs and the possibility to modulate brain inflammation by acting directly on the neurovasculature is intriguing and appealing.

Increased expression of HDACs has been reported in the brain (23) and peripheral white blood cells of MDD patients (38). This change is not unique to depression with alterations also observed in bipolar disorder and schizophrenia, highlighting the important role of these epigenetic enzymes in mood disorders (38, 39). Infusion of HDAC inhibitors exerts robust antidepressant-like effects (7, 15). Here we report a role for hdac1 in stress-induced increase in BBB permeability through repression of *cldn5* expression. Recent sequencing studies performed in whole mouse and human brains revealed that HDAC2 expression is higher than HDAC1 in endothelial cells (14, 40). Here we compare expression of these enzymes in a region-specific manner. Profiles of HDAC expression vary between species (41) and, according to the Allen Brain Atlas, across brain regions as well. Thus, it will be intriguing to compare HDAC2 expression in postmortem samples from depressed patients and manipulate this enzyme in mice to evaluate its potential role in stress responses in future studies. This could be done not only in the NAc but in different brain regions known to be involved in emotional regulation (6). Acetylation is an important posttranslational modification of histones determining accessibility of chromatin and gene transcription (42). In our study we report increased acetylation and reduced methylation of histones at the *cldn5* gene promoter in RES mice giving access to transcription factors to the *cldn5* transcription site. We hypothesize that the reduction in *Foxo1* expression is important to prevent repressive binding and subsequent inhibition of *cldn5* transcription. Conversely, in SS mice lower acetylation is observed when compared to the other groups already limiting accessibility to the *cldn5* promoter for repressive transcription factors. We cannot rule out that changes in transcription factor expression occur at earlier or later time points in SS animals and it will be important to eventually perform longitudinal studies to address this question. It is also worth noting that other transcription factors such as endothelium-enriched SOX18 are known to regulate *cldn5* expression (43, 44) and it will be interesting to evaluate if these factors play a role in stress-induced loss of cldn5 in the future. Despite stress-induced cldn5 loss and lack of change in repressive Foxo1/ctnnb1 expression, the NAc endothelium molecular machinery of SS mice is not adapting properly to compensate when compared to RES animals. This could be directly related to its inflammation. Indeed, proinflammatory NFκB can interact with HDAC1 (Fig. 6), and indirectly with HDAC2 through HDAC1 binding, to repress gene expression (45, 46). Reducing circulating proinflammatory mediators, such as cytokines, represents an attractive therapeutic approach to protect the BBB under stressful conditions (47). It will be interesting to investigate whether treatment with humanized antibodies can prevent stress-induced neurovascular dysfunction.

Despite clinical evidence suggesting a loss of BBB integrity in MDD nearly 35 y ago (10, 47), the cellular and molecular mechanisms have remained poorly understood and causality for inappropriate coping responses under stressful conditions had yet to be confirmed. The availability of novel tools such as high-resolution brain imaging, viral-mediated functional manipulations, and cell-specific transcriptomic analyses finally allows characterization of neurovascular responses in the context of chronic stress and depression in rodents and humans. Interestingly, a recent study showed that self-reported everyday psychosocial stressors are associated with greater impairment in endothelial function in young adults with MDD (48), suggesting that assessment of neurovascular function could help confirm this mood disorder diagnosis. In support of this, reduction of brain-associated markers in blood serum, an indirect indication of BBB integrity, has been shown to be predictive of antidepressant response (49). While the current work was only performed in male mice, depression being far more prevalent in women (50) it will be highly relevant to evaluate whether stress-induced neurovascular adaptations are present in females. In addition, we know that age-related pathologies affecting cognition are highly comorbid with depression. Indeed, BBB breakdown is an early biomarker of human cognitive dysfunction (51) and a link between cldn5 polymorphisms and cognition status in the elderly has been revealed by genome-wide studies of verbal declarative memory in nondemented older individuals (52). A greater understanding of the role of the neurovasculature in stress responses and mood disorders across the lifespan of both sexes could allow for the development of more effective antidepressant strategies, either by augmenting efficacy of current therapies or informing the discovery of new therapeutics. To date, no compound can directly enhance cldn5 expression to prevent or repair endothelial damage; however, the present findings shed light on molecular pathways that could be targeted to promote BBB integrity, neurovascular health, and stress resilience.

## Materials and Methods

### Animals.

Male C57BL/6J mice were purchased at 7 wk of age (∼25 g) from The Jackson Laboratory or Charles River Canada and allowed to rest for 1 wk at either the Icahn School of Medicine at Mount Sinai or CERVO Brain Research Center housing facilities. Sexually experienced retired CD-1 male breeders (∼40 g) were used as aggressors (AGGs) and purchased from Charles River. All mice were singly housed following chronic social stress until tissue collection and maintained on a 12-h light/dark cycle with ad libitum access to water and food. All experiments were conducted in the dark phase and performed according to the National Institutes of Health Guide for Care and Use of Laboratory Animals and the Canadian Council on Animal Care as well as animal care and use committees of the Icahn School of Medicine at Mount Sinai or Université Laval.

### CSDS.

The CSDS paradigm was performed as described in Golden et al. (16). Briefly, CD-1 mice were screened for aggressive behaviors for 3 consecutive days, then housed on one side of a clear perforated Plexiglas divider (0.6 cm × 45.7 cm × 15.2 cm) in social defeat cages (26.7 cm width × 48.3 cm depth × 15.2 cm height, Allentown Inc.) 24 h before the first defeat session. Experiment C57BL/6J mice were subjected to physical interaction with an unfamiliar CD-1 AGG for up to 10 min once per day for 10 consecutive days (Fig. 1*A*). At the end of the physical encounter, experimental mice were removed and housed on the opposite side of the social defeat cage divider to allow sensory interactions for the subsequent 24 h. C57BL/6J mice are rotated each day to be exposed to a novel unfamiliar AGG for each physical interaction session. Unstressed control mice were housed two per cage in a social defeat cage with a divider and rotated daily in a similar manner without ever being exposed to a CD-1 AGG mouse.

### SI Test.

The SI test was performed under red light as described in Golden et al. (16) to establish behavioral phenotypes of stressed mice. It consists of two sessions of 2.5 min each. First, experimental mice were placed in an open-field arena (42 cm × 42 cm × 42 cm, Nationwide Plastics) including a small wire animal cage placed in the middle of a wall at one end. Movements were automatically monitored and recorded with a tracking system (Ethovision 11.0, Noldus Information Technology) to determine baseline exploratory behaviors and locomotion in absence of an unfamiliar social target. At the end of the 2.5 min, the experimental mouse was removed, and the arena was thoroughly cleaned. In the second session, a novel CD-1 mouse was placed in the small wire animal cage and the experimental mouse was allowed to explore the arena once more. Time spent in the interaction zone, surrounding the small wire animal cage, corners, and overall locomotion was compared to unstressed CTRL. SI ratio was calculated by dividing the time spent in the interaction zone when the AGG is present divided by the time when the AGG is absent. Stressed mice with a ratio below 1.0 were classified as SS while mice with a ratio above 1.0 were considered RES.

### Mouse Chromatin Immunoprecipitation.

Site-directed quantitative chromatin immunoprecipitation (qChIP) was performed as described in Golden et al. (15) to produce fragments of 350 to 700 bp in length. Briefly, following 10 d of CSDS and SI test, brains were removed and bilateral NAc was collected from two adjacent 1-mm coronal slices with 14-gauge needles. Punches were immediately cross-linked for 15 min at room temperature in 1% formaldehyde and quenched by adding 0.125 M glycine. After five washes in cold PBS solution containing protease inhibitors punches were frozen on dry ice. Each sample includes 8 NAc punches from two mice balanced by SI ratio. Next, chromatin was solubilized, extracted by two steps of 7-s sonication at low power in 500 μL SDS-detergent lysis buffer (1% SDS in 50 mM Tris⋅HCl, 10 mM EDTA pH 8.1) and diluted in 1,100 μL of ChIP dilution buffer (16.7 mM Tris⋅HCl, pH 8.1m with 167 mM NaCl, 1.2 mM EDTA, 0.01% SDS, 1.1% Triton X-100) before five steps of 15-s sonication at high power. A total of 400 μL of sheared chromatin was used for each IP and diluted to a final volume of 600 μL in ChIP dilution buffer. ChIP was performed with 7 μg of anti-acetyl H3 and anti-H3K27me3 antibodies (EMD Millipore) per sample, conjugated to magnetic Dynabeads (M-280 sheep anti-rabbit IgG, Invitrogen). Beaded antibodies were incubated with the samples overnight at 4 °C, washed eight times in ChIP RIPA buffer, then the beads were removed by a 30-min heating step at 65 °C (with shaking at 1,000 rpm). Supernatant was collected by spinning and cross-linking of chromatin in the supernatant and input samples were reversed by heating overnight at 65 °C. DNA was purified for quantitative PCR analysis with a QIAquick PCR purification kit (Qiagen). Mapping of the chromatin landscape at the *cldn5* promoter was done with the primers listed in *SI Appendix*, Fig. S2.

### Human Postmortem Tissue Collection.

Whole-tissue NAc sections for qPCR analyses and immunostaining were collected and provided, for the Montreal cohort, by the Douglas-Bell Canada Brain Bank (Douglas Hospital Research Center) under approval of the institution’s research ethics board (REB) (7, 15). For *CLDN5* promoter epigenetic characterization, whole-tissue NAc sections were collected and provided by the Dallas Brain Collection (tissue was collected by the Dallas Medical Examiner’s Office and the University of Texas Southwestern Institutional Review Board) (7, 15). All experiments were performed under the approval of Université Laval and CERVO Brain Research Center Ethics Committee. Brain tissue was collected at the local medical examiner’s office and blood toxicology performed after obtaining permission to exclude subjects with illicit drug use or psychotropic medication. Subjects with a known history of head injury or neurological disorders were excluded as well. Demographic characteristics of each cohort are listed in *SI Appendix*, Table S3. Independent diagnosis was established in line with the Diagnostic and Statistical Manual of Mental Health Disorders (DSM) IV criteria via clinical records and interviews by three or four mental health professionals. Cohorts were matched as closely as possible for gender, age, race, pH, postmortem interval, and RNA integrity number (7, 15).

### Human Chromatin Immunoprecipitation.

Again, site-directed qChIP was performed as described in Golden et al. (15) this time using a micrococcal nuclease (MNase)-based assay to produce fragments of ∼150 to 160 bp. First, 50 mg of human NAc was homogenized in 550 μL douncing buffer (10 mM Tris⋅HCl, pH 8.0 with 4 mM MgCl2 and 1 mM CaCl2) in a glass homogenizer. Homogenates were then digested with the MNase enzyme (2 units/mL) at 37 °C for 10 min in a water bath and the reaction stopped by adding 10 mM EDTA, pH 8.0. Digested chromatin was incubated in SDS-detergent lysis buffer for 60 min at 4 °C with agitation every 10 min. To collect the supernatant, lysed chromatin was centrifuged at 3,000 × *g* for 20 min at 4 °C. A total of 400 μL of digested chromatin was used for each ChIP in a final volume of 500 μL completed with incubation buffer (200 mM Tris⋅HCl, pH 8.0, 500 mM NaCl, 50 mM, EDTA). ChIP was performed with 7 μg of anti-H3K27me3 antibodies (EMD Millipore) per sample conjugated to magnetic Dynabeads (M-280 sheep anti-rabbit IgG, Invitrogen). Beaded antibodies were incubated with IP chromatin overnight at 4 °C, washed eight times in ChIP RIPA buffer, then beads were removed by a 30-min heating step at 65 °C (with shaking at 1,000 rpm). Supernatant was collected by spinning and chromatin in the supernatant and input samples were reverse cross-linked by heating overnight at 65 °C. DNA was purified for quantitative PCR analysis with a QIAquick PCR purification kit (Qiagen). Level of H3K27me3 methylation at the *CLDN5* promoter was done with the primers listed in *SI Appendix*, Table S1.

### RNAscope.

Mice were anesthetized with a mixture of ketamine (100 mg/kg of body weight) and xylazine (10 mg/kg of body weight), perfused for 7 min with 0.1 M PBS and the brains quickly extracted and frozen in optimal cutting temperature (OCT) compound using isopentane on dry ice (7). Blocks were stored at −20 °C for 24 h before slicing on a cryostat at 12-μm thickness. Slices were thawed, mounted onto Superfrost Plus (Fisher Scientific) slides, and then stored at −80 °C for future analysis. In situ hybridization was performed using the RNAscope system (Advanced Cell Diagnostics). First, samples were postfixated in 4% paraformaldehyde solution, followed by three-step dehydration with 50 to 100% ethanol solution and pretreatment protocol of 15 min with Protease III at room temperature. Next, probe hybridization with the RNAscope Fluorescent Multiplex Kit was performed according to the manufacturer’s protocol. Probes included CD-31 (316721-C1), *FoxO1* (485761-C2), and *ctnnb1* (311741-C3). Confocal fluorescent images of the NAc shell and core regions were captured with a Zeiss Lsm 700 microscope using a 63× objective (Zeiss PLAN-APOCHROMAT, numerical aperture = 1.3) with oil immersion. Cell bodies stained with 4,6-diamidino-2-phenylindole (DAPI) and RNA clusters were identified using a homemade software in Matlab (MathWorks). Bright cell bodies were identified by calculating a local average and local SD of the images (subregion of 200 × 200 pixels). Pixels with intensities higher than the mean plus 0.5 to 1 SD were selected. A morphological analysis on the selected clusters was applied and objects that scored a size higher than 500 pixels and maximum eccentricity of 0.9 were kept. Detection of the RNAscope clusters of *CD31* and *ctnnb1* were drawn manually by the user using the imellipse function in Matlab. Detection of *CD31* and *Foxo1* was done automatically with the same algorithm as the DAPI identification but with a local average and SD of 15 × 15 pixels. Bright pixels of *CD31* and *Foxo1* with intensities higher than the mean plus 2.5 SD, size between 7 and 200 pixels and eccentricity higher than 0.97 were kept. The number, area, and intensity of each RNA cluster in each channel was measured on the DAPI-positive cells.

### MACS of Endothelial Cells.

NAc samples were collected following behavioral assessment as described previously (7). Bilateral 14-gauge punches were collected from two adjacent 1-mm coronal slices on wet ice after rapid decapitation and immediately processed for MACS purification. Endothelial cells were enriched from NAc punches by using MACS according to the manufacturer’s protocol (Miltenyi Biotec). Briefly, brain punches were dissociated using a neuronal tissue dissociation kit (Miltenyi Biotec, 130-092-628), applied on a 70-μm MACS smart strainer and washed with HBSS 1×. Thereafter, cells were magnetically labeled with CD45 microbeads (Miltenyi Biotec, 130052301) and passed through a MACS MS column (Miltenyi Biotec, 130-042-201) to proceed to negative selection of CD45 cells. CD45^−^ fraction was collected and magnetically labeled with CD31 microbeads (Miltenyi Biotec, 139097418) and then passed through MACS MS column to positively select CD31^+^ cells. CD45^−^, CD31^+^ cells were resuspended in 200 μL of TRIzol for RNA extraction and transcriptome-wide gene-level expression profiling. Small aliquots of each fraction were used for flow cytometry assessment of endothelial cell enrichment.

### Affymetrix Clariom S Transcriptome-wide Gene-Level Expression Profiling.

Samples were shipped to Genome Quebec for RNA extraction, quality control with the Bioanalyzer, and gene expression analysis with the Affymetrix Clariom S Pico assay for mouse (Thermo Fisher Scientific). Gene expression analysis was performed with the Transcriptome Analysis Console 4.0 provided by Thermo Fisher with the Clariom S assay according to the manufacturer’s instructions. To identify significant changes between groups, filters were set at fold change ±2 and *P* < 0.05.

### Flow Cytometry of Endothelial Cells.

Original, CD45^−^, CD45^+^, CD45^−^ CD31^−^, and CD45^−^ CD31^+^ cell fraction aliquots obtained following MACS separation were incubated with anti-CD16/32 (BioLegend, 14-0161-82) to block Fc receptors. Cells were then labeled with CD45 APC (BioLegend, 103111) and CD31 PE-CF594 (BD Biosciences, 653616). A viability dye (LIVE/DEAD fixable green, Molecular Probes, L34969) was added to the previous panels to discriminate live cells. Endothelial cells from mouse brain NAc punches were identified as CD45^−^ and CD31^+^ cells. All analyses were performed on BD LSR II and data were analyzed with FACS Diva software (BD Biosciences).

### Transcriptional Profiling by qPCR.

NAc samples were collected and processed as described in Menard et al. (7). Bilateral 14-gauge punches were collected from 1-mm coronal slices on wet ice after rapid decapitation and immediately placed on dry ice and stored at −80 °C until use. RNA was isolated by TRIzol homogenization, chloroform layer separation, and the clear RNA layer was processed with a RNeasy Micro Kit (Qiagen) before quality control and quantification on a NanoDrop (Thermo Fisher Scientific). A total of 500 ng of RNA was reversed transcribed to cDNA with qSCRIPT (Quanta Biosciences) and resulting cDNA was diluted to 500 μL. A total of 3 μL of cDNA was used for each quantitative PCR with 0.5 μL of forward and reverse primers or 1 μL of PrimeTime qPCR primers (IDT Integrated DNA Technologies), 1 μL of water, and 5 μL of Perfecta SYBR Green (Quanta Biosciences). Samples were heated to 95 °C for 2 min followed by 40 cycles of 95 °C for 15 s, 60 °C for 33 s, and 72 °C for 33 s. Analysis was done with the ΔΔCt methods and samples were normalized on *gapdh* or *GAPDH* housekeeping genes for mouse or human samples, respectively. Primer pairs are listed in *SI Appendix*, Table S2.

### Pharmacological Treatment with MS-275 hdac Inhibitor.

SS mice were anesthetized with a mixture of ketamine (100 mg/kg) and xylazine (10 mg/kg) and surgically implanted with two subcutaneous Alzet minipumps (model 1002, Durect) and bilateral cannulae targeting the NAc as described in Golden et al. (15). Minipumps and cannulae were filled with MS-275 (100 μm, provided by the Broad Institute, Cambridge, MA) or 5% hydroxypropyl β-cyclodextrin as vehicle (Trappsol, CTD, Inc.) the day before the surgery. An incision was performed over the skull and the skin spread apart under the scapulae to create a space to position the minipumps on the back. Bilateral cannulae were delivered into the NAc at the following coordinates according to Bregma: anteroposterior, +1.5; mediolateral, +1.0; dorsoventral: −4.5, and fixed to the skull with Loctite skull adhesive (Henkal). Tubing and minipumps were fixed under the skin with Vetbound tissue adhesives and staples.

### Immunofluorescence.

Human tissue slides were removed from a −80 °C freezer, postfixed in ice-cold methanol for 10 min, and briefly washed in 0.1 M PBS. Sections were incubated for 2 h in 5% normal donkey serum (NDS) and 0.1% Triton X-100 (Fisher Bioreagents, BP151-100) in 0.1 M PBS for 2 h, before being incubated overnight at 4 °C with primary antibodies (rabbit anti-Cldn5, 1:250, Thermo Fisher Scientific, 34-1600 and sheep anti-hCD31, 1:250, R&D Biosystems, AF806). Slices were washed three times in 0.1 M PBS for 5 min and incubated with anti-rabbit Cy2 and anti-sheep Cy3 secondary antibodies for 2 h at room temperature (1:400, Jackson Immunoresearch, 711-225-152, 713-165-147, respectively). Sections were again washed three times in 0.1 M PBS and counterstained with DAPI to visualize nuclei. Slices were mounted and coverslipped with ProLong Diamond Antifade Mountant (Invitrogen, P36961). Images were acquired from 6-μm flattened z-stacks on a LSM-700 confocal microscope (Carl Zeiss) using a 20× lens with a resolution of 512 × 512 and a zoom of 1.0.

### Statistical Analysis.

Sample size was calculated based on previous studies (7, 15). Outliers were identified as being greater than 2 SD from the mean and excluded from statistical analyzes. Mice were assigned to SS or RES subgroups based on their behavioral profile when compared to CTRL. SI tests were performed with automated tracking. For the epigenetic experiment, mice were pooled based on their SI ratio to obtain comparable average values between each sample of a subgroup. RNAscope and MACS analyzes were performed with a custom automated program using MATLAB software and Thermo Fisher Transcriptomic Analysis Console, respectively. For the pharmacological experiment, CTRL and SS mice were randomly assigned to vehicle- or MS-275–treated groups. All *t* tests, one-way ANOVAs, two-way ANOVAs, and Pearson’s correlation were made with GraphPad Prism software (GraphPad Software Inc.) with statistical significance set at *P* < 0.05. Bonferroni posttest was used as a post hoc test when appropriate for one-way or two-way ANOVA and statistical significance set at *P* < 0.05.

### Data Availability Statement.

Raw sequencing data are available in the Supporting Information. Protocols and code are available by contacting the corresponding author and on the Menard Neuro Lab website (http://menardneurolab.com).

## Supplementary Material

Supplementary File

Supplementary File
