# Supplementary material for: Molecular adaptations of the blood–brain barrier promote stress resilience vs. depression
Source: Proc Natl Acad Sci U S A. 2020 Jan 23;117(6):3326–36. doi: 10.1073/pnas.1914655117 (PMC7022213; doi:10.1073/pnas.1914655117)
Supplement: Supplementary File [file pnas.1914655117.sapp.pdf]

Molecular adaptations of the blood-brain barrier promote stress resilience vs depression

Katarzyna A. Dudek[1], Laurence Dion-Albert[1], Manon Lebel[1], Katherine LeClair[2], Simon Labrecque[1], Ellen Tuck[1,3], Carmen Ferrer Perez[2,4], Sam A. Golden[2,5], Carol Tamminga[6], Gustavo Turecki[7], Naguib Mechawar[7], Scott J. Russo[2], Caroline Menard[1]*

[1]Department of Psychiatry and Neuroscience, Université Laval and CERVO Brain Research Center, 2601 de la Canardiere, Quebec City, QC (Canada), G1J 2G3;
[2]Nash Family Department of Neuroscience, Icahn School of Medicine at Mount Sinai and Center for Affective Neuroscience, 1 Gustave L Levy Place, New York, NY (USA), 10029-5674;
[3]Smurfit Institute of Genetics, Trinity College Dublin, Lincoln Place Gate, Dublin 2 (Ireland);
[4]Department of Psychobiology, University of Valencia, Av. De Blasco Ibanez, Valencia (Spain), 46010;
[5]Department of Biological Structure, University of Washington, 1959 NE Pacific Street, Seattle, WA (USA), 98195;
[6]Department of Psychiatry, the University of Texas Southwestern Medical Center, 5323 Harry Hines Blvd, Dallas, TX (USA), 75390;
[7]Department of Psychiatry, McGill University and Douglas Hospital Research Centre, 6875 Blvd LaSalle, Montreal, QC (Canada), H4H 1R3.

Corresponding author: **Caroline Menard,** PhD

CERVO Brain Research Center
Department of Psychiatry and Neuroscience
Faculty of Medicine, Université Laval
2601 de la Canardiere
Quebec City, QC, Canada
G2J 2G3

(418) 663-5741

E-mail: caroline.menard@fmed.ulaval.ca

Key words: mood disorders, antidepressant, vascular, inflammation, epigenetic

www.pnas.org/cgi/doi/10.1073/pnas.1914655117

Supplementary Figures and Legends

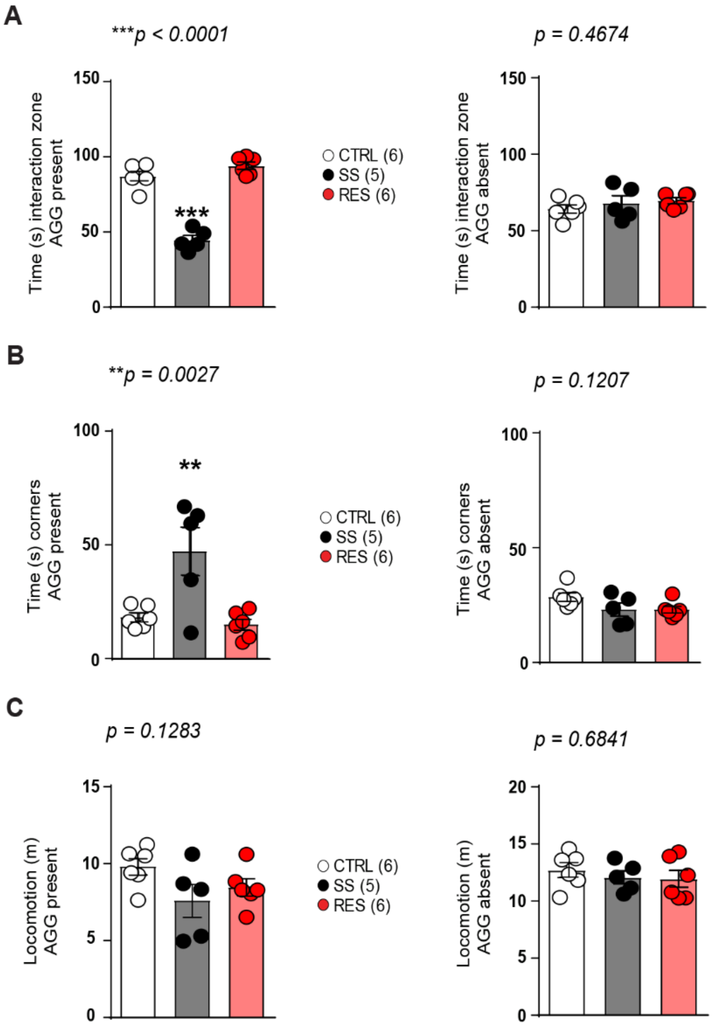

Supp. Fig.1 – Extended behavioral data of social interaction test for cldn5 epigenetic regulation. Stress-susceptible (SS) mice spent less time in (**A**) the interaction zone (one-way ANOVA: $F_{2,14} = 79.69$; ***$p < 0.0001$) and more time in (**B**) the corners (one-way ANOVA: $F_{2,14} = 9.295$; **$p = 0.0027$) when the aggressor (AGG) was present (left) vs absent (right) when compared to unstressed controls (CTRL) and resilience (RES) mice (n = 10-12 mice/group). **C**) No significant difference was observed for locomotion (one-way ANOVA: $F_{2,14} = 2.387$; $p = 0.1283$).

Forward primer
Reverse primer

Mouse CLDN5 promoter, chromosome 16: 18,774,799-18,776,834

3494 ....actgagactatattttggagaggtcaggctggactgttgtgggcctaaaagggatggtggggctacaggaccccaccctctcaaaactctcctgctcactgaa 3391

3390 gcacagtggcctctgatattgatggctagatggcacctgcttgggcctgaggtccatgaactgtgaaggtcaactgagctagtgagtaacttcaagcccctctc.... 3281

3073 ....ggagggggtcttaagatgcttcctgttgggccagtgagagcctgggctttttgtcttccaccaagaaatggctctgggcaagaagaatgttgttgacctgtaaa 2969

2968 tgtctgctgaacttggggaagacatcctggaccagaaactgaaagatagaagtaagctggggtttttgtttttgtttttgtttccttccctcactcccttgaaataaag... 2854

2425 ...gcatctgtacaccagcatcttatactcagatttccaactgctccttgggatcttcttcctggactcctgctgcgctgccaccttttcagagaaccctctgctgggttttg 2316

2315 ccttcctatctggtactttgttggcagtttgtttattaagtggcctgtgctgtgtgctctgggagagacagtgtccaatgcacacacacacacacacacacacac...... 2205

2102 ...atttctggagtggttctggtcccgctgcatgctggagagtacggtatctaaggttccactggcaattatgaatttaccaaaggtatgtgaacttgctcggaccattct 1995

1994 gggtgcaaggctggggacagtaccgaaatggtgggtaggagggcagacagaaggaagggagcatgtcctgctgtttgtagccccaaatagttgaaagca 1896

1985 gcaagctgcttcttgtctgggacaaatgtgtgtcccagaagatccccgtattcccgggacactagtaggaaagccagcgggcaaaagctatcctggcttgga.... 1790

1580 ..aatgagagccatagaggattacaggccagggcctgcaggagtagccttgttgggtcatctgaagagggttcagtagttgctccaggaaggtgcctgggagctt 1478

1477 gaagggagggggctggcaaaacaaaattctgcagactggagcaggggggagcaggatttgagtttacatctactgagcagggagaagggtctgcagaaaag 1378

1377 gcaggggtctctagaagatagaaggcagcagttaatatatgatagctggaagaggtaccagggaggacaagaaagggtgtgtatgtgaggctctcccgccgt 1277

1276 cttctaagaacagtttgttgtagggtgaaagtaggcaatgacctggccccatagacctctagctcagagccatctccagaaggaagcagggacccataaaggg 1174

1173 agagatttaccaaaatctccacatctgtggctggcctcaacaatgtcatcctttccctatagaggccctgagttcttttgagcagaggaaacaacaaaacaaa 1071

1070 acccccaaacagaacaacaacagaagacaacaaagcccagctggagtccagggttagctggaaacaaagggagcagggaggagtgttcactgga..... 971

762 ....cctggggggctggggtggcaaagcaccacaactatttgtaggatggcaaggcatagcctttctatgtcccatcgtgtctctgctgtcccaggattccagtatgctgt 657

656 gttaggatcacagcctatctgctggagaagactggacgtcggagaaccctcaggggttgcgcagcatcccagaggagagcacaggcccttgcagtgttcacttt 553

552 gtgtcagaccagtggttccagggaaggagtgagaactgtaggcggtggttgggtaggatcatgaatgaggttataattaagggcaagcccaacacaggcttgc 450

449 cgagcatctccgcttggtgtgccaagggaaaaacggcgggcgtagaggtttgtttcacctgtgcacgggggctcagagaacctccaggccaagcagtctgcag 347

346 agaggagttggaatgatccgggatgtagcaggtggagctggtgagtgtaggttaggggtcatgctgctaacagtggagacaggggcgcacagatctcctgtgta 243

242 cctgccaagatcagcccctactaggacagaaactggtagggtctcagactgcggttctgcagggtgcggttcgggtgagcaggactagtcctgctcgcccgt.... 137

-972 ...gggccctgctcagaacagactacaggcacttttaagaacttgaccgaccttttcttctatgcgcagttggccacgacgtgggtggaacgctcagatttcatcgg -1076

-1075 tgaagtaggcaccaaactgccgcgaacagttcctactgagatcctgggggcactagatgctgccttaatgtccagtggcacctgctaacctgaaagggca... -1176

Supp. Fig.2 – Cldn5 primer sequence for mouse epigenetic studies. Pair of primers were designed to cover the transcriptional site up to ~3500bp before start of the transcriptional site according to (1) who characterized for the first time *cldn5* promoter region.

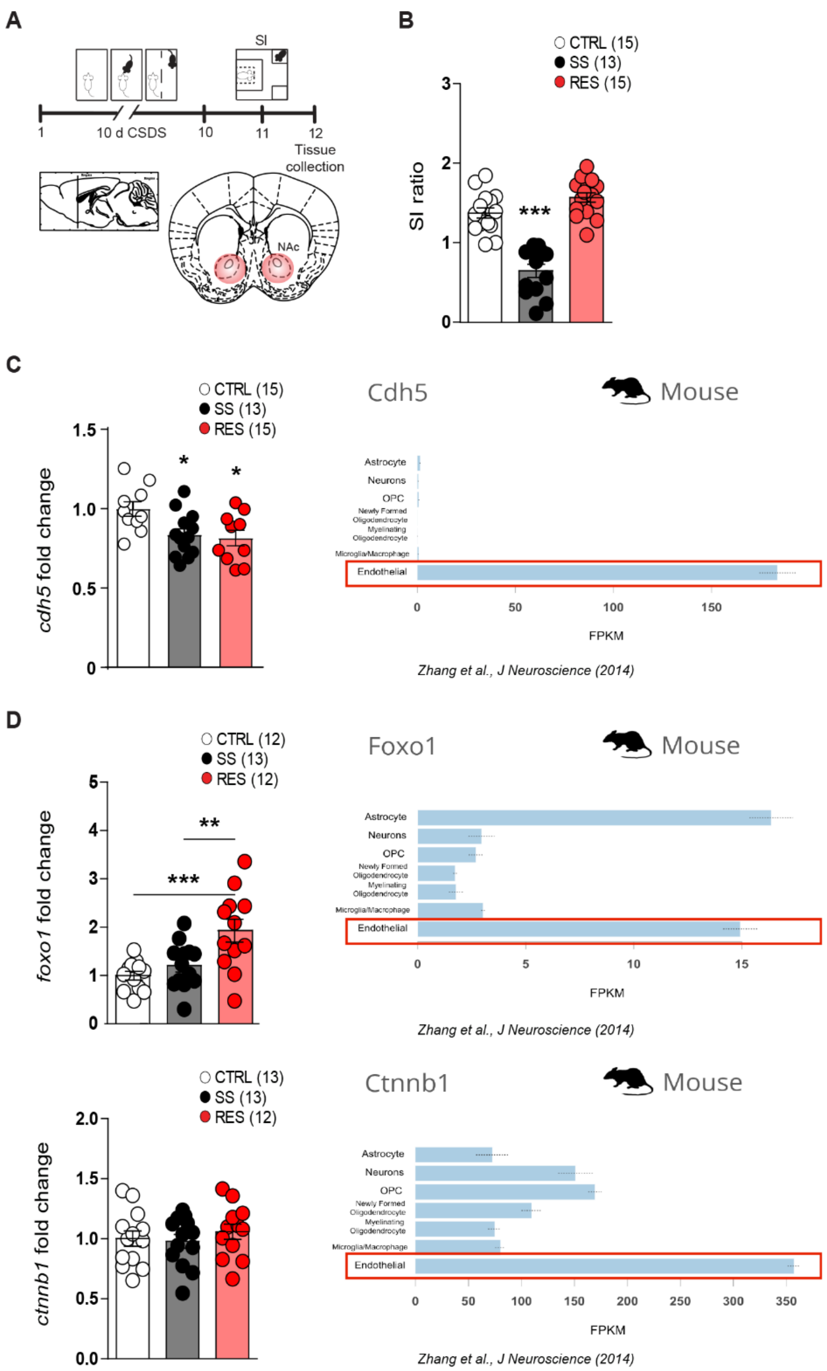

Supp. Fig.3 – Stress-induced changes in NAc cldn5-related cell signaling genes. A) Experimental timeline and (**B**) SI ratio of CTRL, SS and RES mice for NAc quantitative PCR analysis (one-way ANOVA: $F_{2,40} = 48.03$; ****p* < 0.0001). **C**) Endothelial cell-specific *cdh5* is reduced in both SS and RES mouse subpopulations (one-way ANOVA: $F_{2,30} = 4.935$; **p* = 0.0140, n=13-15 mice/group). **D**) *FoxO1* expression is increased in the NAc of RES mice (one-way ANOVA: $F_{2,34} = 8.827$; ****p* = 0.0008, n=12-13 mice/group) while no change was observed for *ctnnb1* (one-way ANOVA: $F_{2,35} = 0.4362$; *p* = 0.6500, n=12-13 mice/group) (**E**).

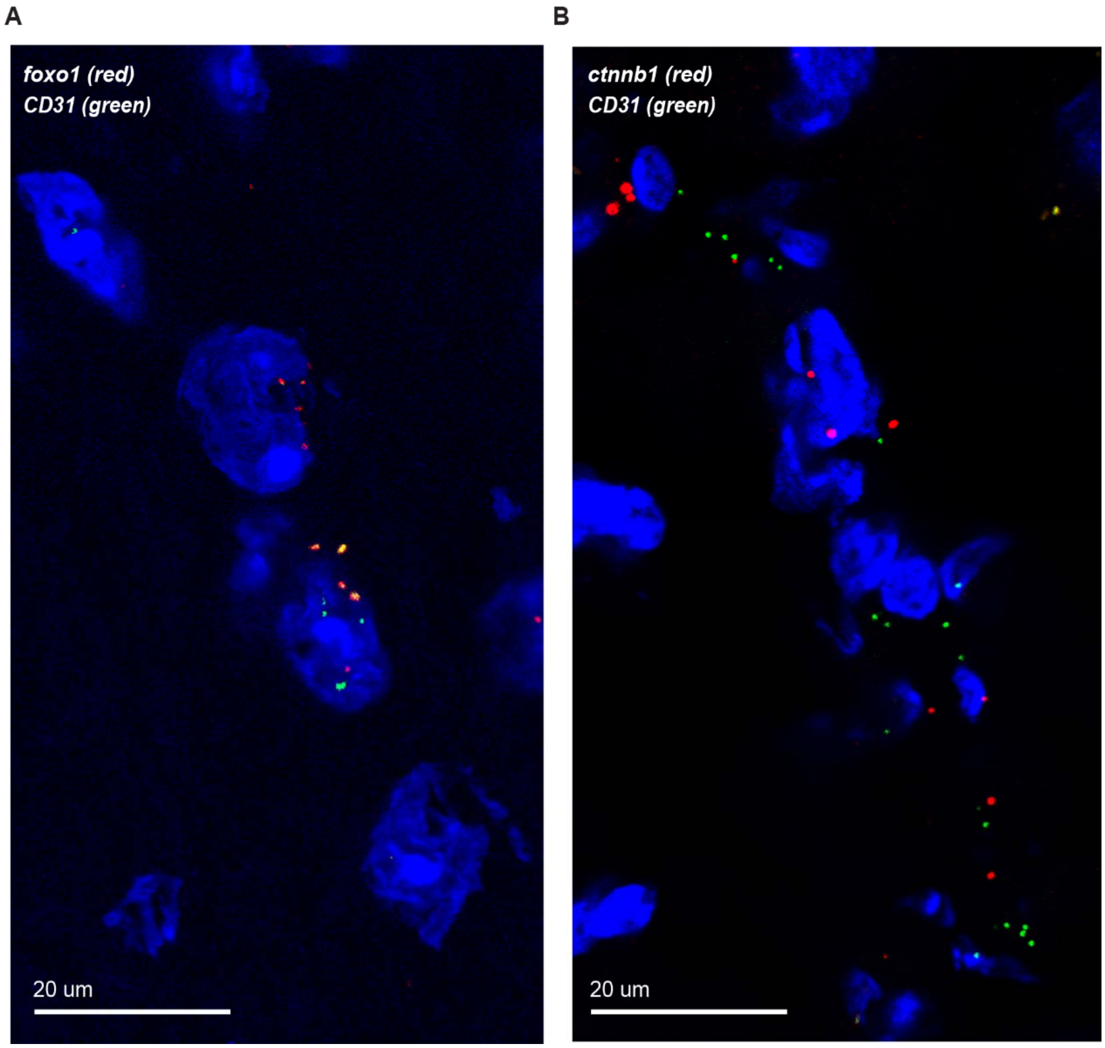

Supp. Fig.4 – Vessel double stained with RNAscope for CD31 endothelial cell marker and cldn5-related transcription factors. Representative vessel imaged at lower magnification for CD31 and *foxo1* (**A**) or *ctnnb1* (**B**). Scale bar set at 20 um.

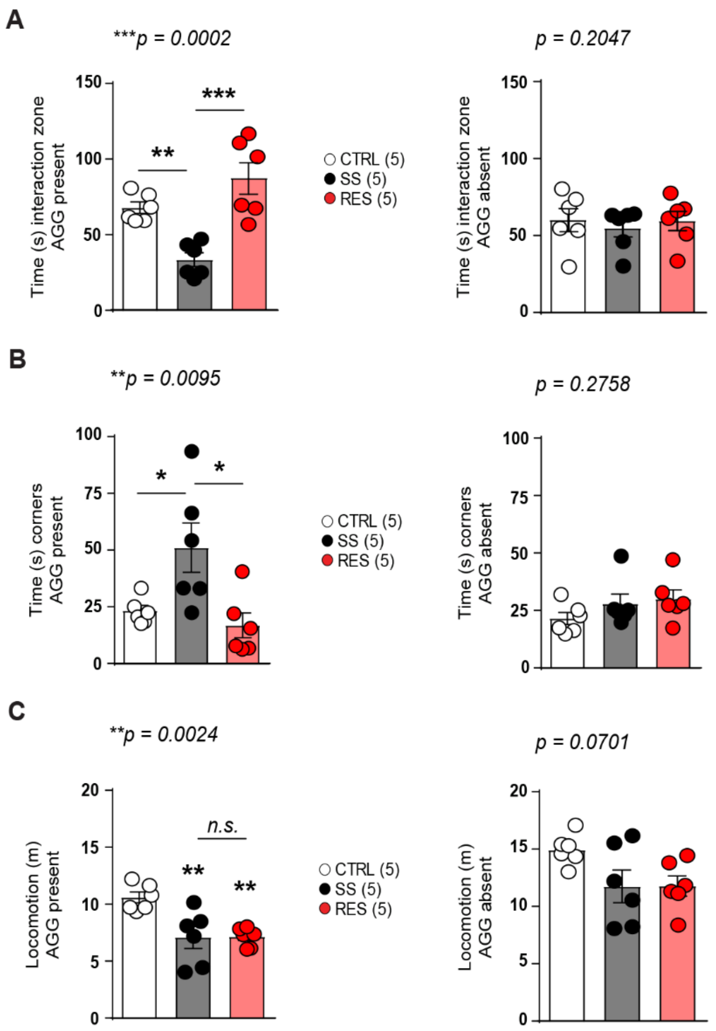

Supp. Fig.5 – Extended behavioral data of social interaction test for endothelial cell-specific transcription factor expression. A) Stress-susceptible (SS) mice spent less time in the interaction zone (one-way ANOVA: $F_{2,15} = 15.38$; ***$p = 0.0002$) and (**B**) more time in the corners (one-way ANOVA: $F_{2,15} = 6.447$; **$p = 0.0095$) when the aggressor (AGG) was present (left) vs absent (right) when compared to unstressed controls (CTRL) and resilience (RES) mice (n = 10-12 mice/group). **C**) No significant difference was observed for locomotion between SS and RES subpopulations, but stressed mice were characterized by lower distance travelled when compared to unstressed controls (one-way ANOVA: $F_{2,15} = 9.293$; **$p = 0.0024$).

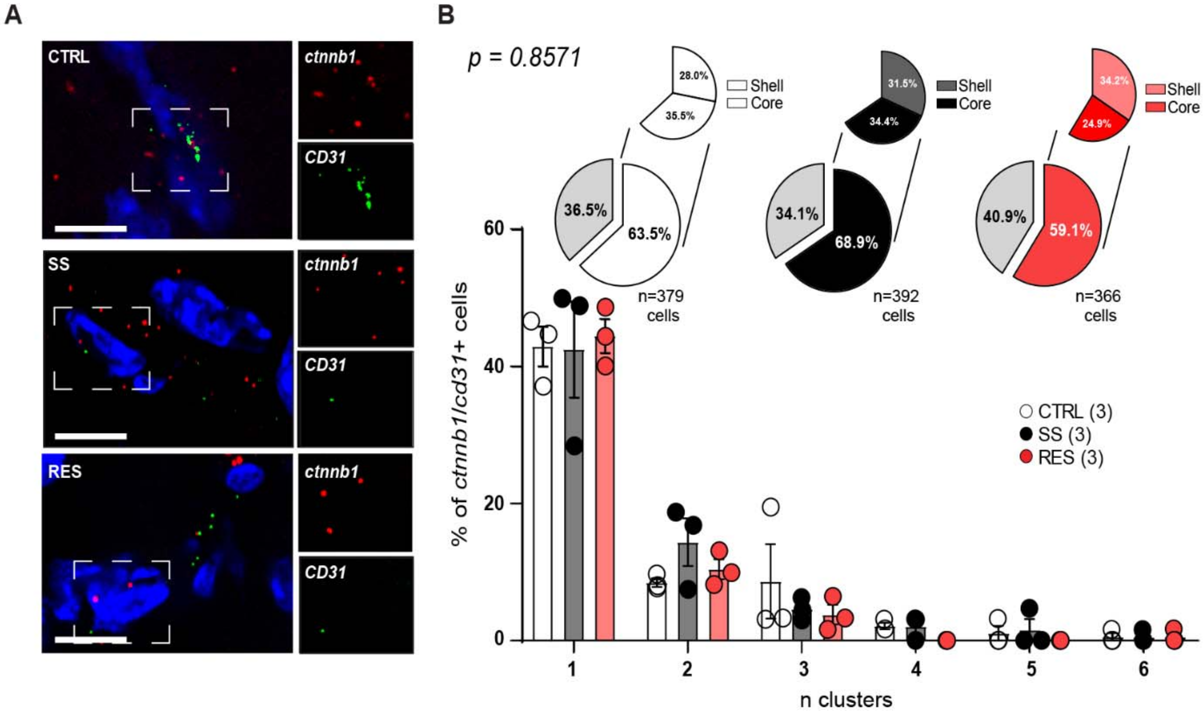

Supp. Fig.6 – Expression of repressive cldn5-related transcription factor *ctnnb1* is unchanged in the endothelium under chronic stress conditions. A) No significant difference was observed for *ctnnb1* expression (two-way ANOVA: phenotype x n clusters interaction effect $F_{10,36} = 0.5303$; $p = 0.8571$, n=366-392 cells from 3 mice/group) (**B**). Non-endothelial cells are identified in light grey in the pie charts and endothelial cells, double labeled with *CD31*, split between shell (white for CTRL, dark grey for SS, pink for RES) and core (white for CTRL, black for SS, red for RES) subregions of the NAc.

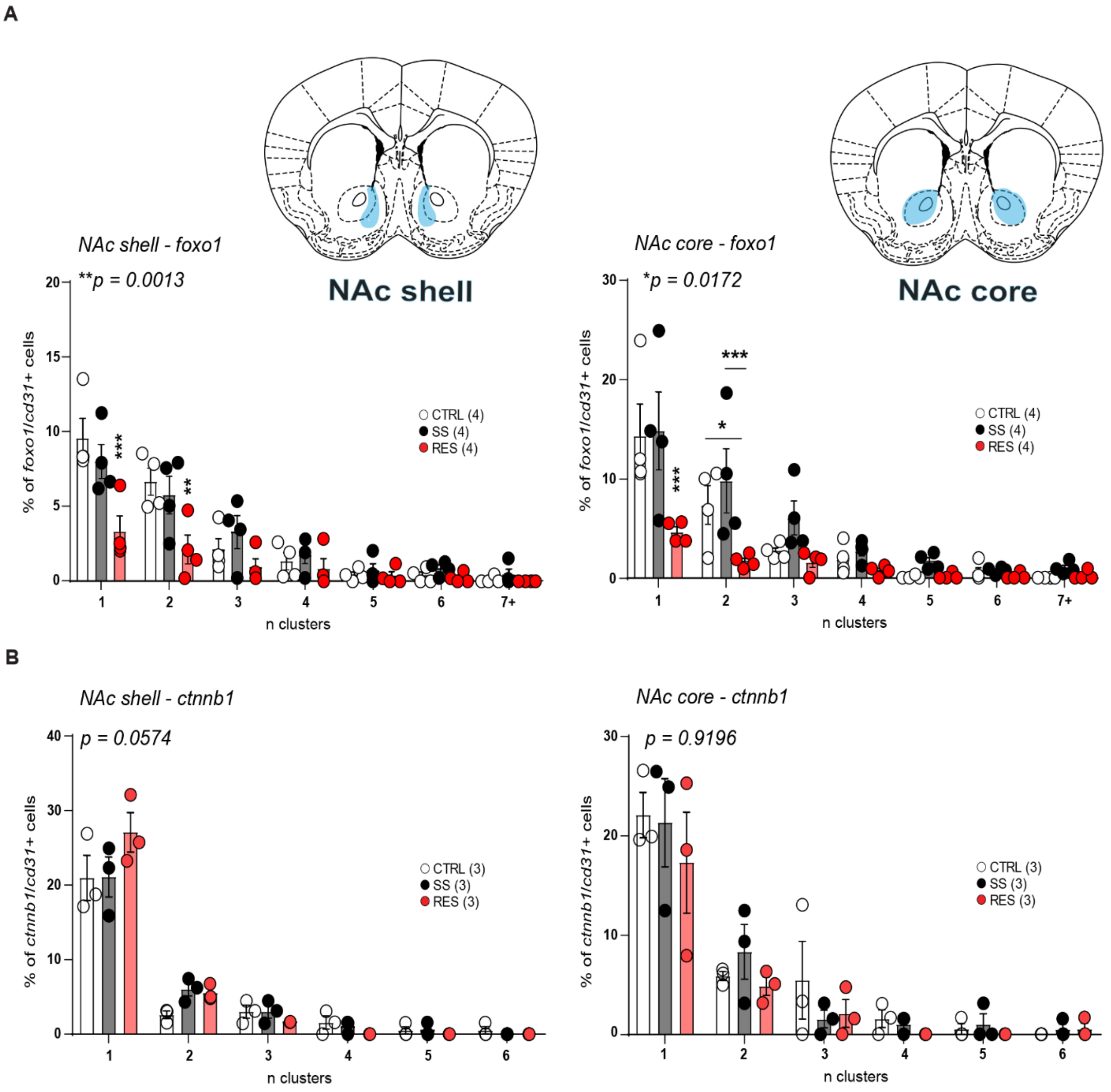

Supp. Fig.7 – Expression of cldn5-related transcription factors in NAc shell vs core subregions. A) Quantification of double labeled *FoxO1/CD31* positive cells revealed significant changes in both shell (two-way ANOVA: phenotype x n clusters interaction effect $F_{12,63} = 3.189$; $***p = 0.0013$) and core (two-way ANOVA: phenotype x n clusters interaction effect $F_{12,63} = 2.29$; $*p = 0.0172$) NAc subregions in RES mice. **B**) A trend was observed for double labeling of ctnnb1/*CD31* in the NAc shell (two-way ANOVA: phenotype x n clusters interaction effect $F_{10,36} = 2.042$; $p = 0.0574$), but no significant difference was measured in the core (two-way ANOVA: phenotype x n clusters interaction effect $F_{10,36} = 0.4346$, $p = 0.9196$).

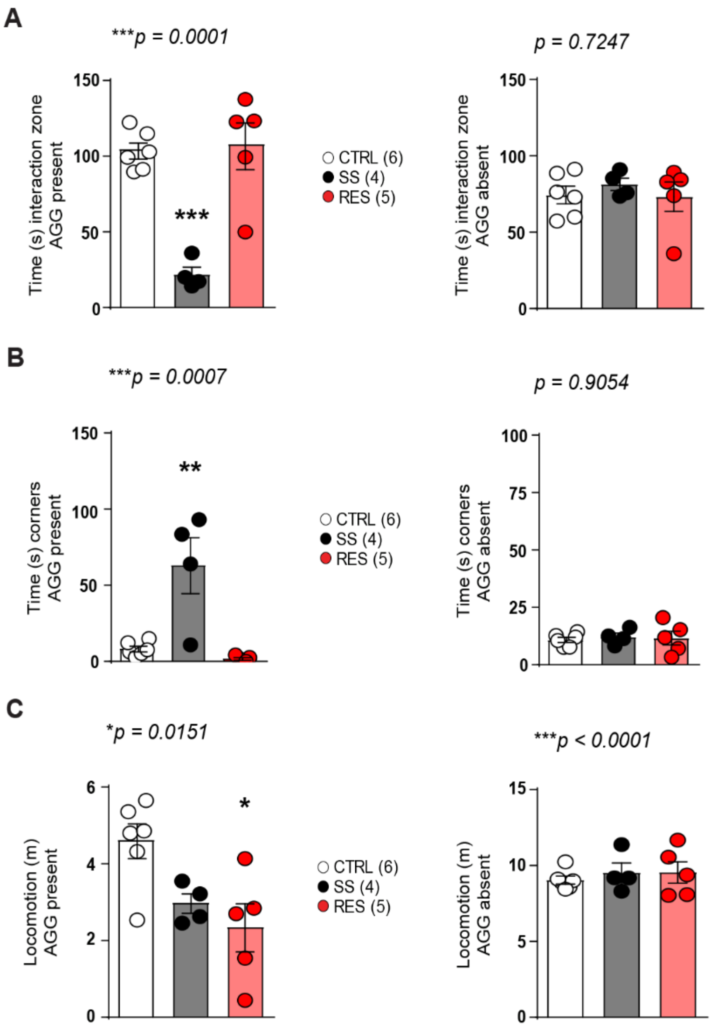

Supp. Fig.8 – Extended behavioral data of social interaction test for magnetic activated cell sorting and mouse Clariom S assay. A) Stress-susceptible (SS) mice spent less time in the interaction zone (one-way ANOVA: $F_{2,12} = 20.58$; ***$p = 0.0001$) and (**B**) more time in the corners (one-way ANOVA: $F_{2,12} = 14.19$; ***$p = 0.0007$) when the aggressor (AGG) was present (left) vs absent (right) when compared to unstressed controls (CTRL) and resilience (RES) mice (n = 10-12 mice/group). **C**) No significant difference was observed for locomotion between SS and RES subpopulations, but RES mice were characterized by lower distance travelled when compared to unstressed controls (one-way ANOVA: $F_{2,12} = 6.062$; *$p = 0.0151$).

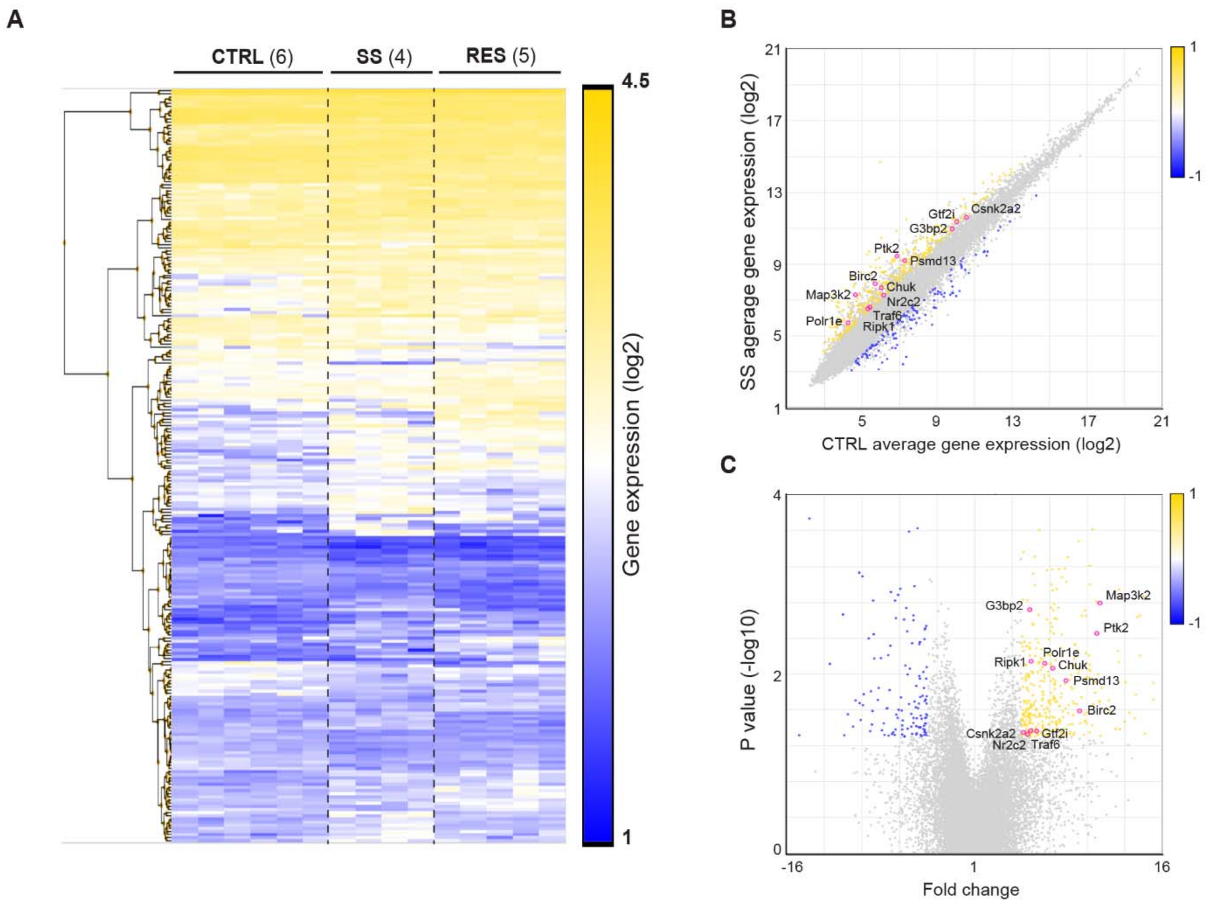

Supp. Fig.9 – Stress-induced transcriptome-wide changes in NAc endothelial cell gene expression and genes associated with stress susceptibility. A) Heat map of gene expression changes in the NAc endothelial cells of CTRL, SS and RES mice. Significance was set at +/- 2-fold change and $p < 0.05$. **B**) Expression of 12 genes associated with the TNFα/NFκB signaling pathways are significantly increased in SS mice when compared to unstressed CTRL. **C**) Fold change of these genes ranges between 2.06 and 6.38 with p-values of *Map3k2*: ***p* = 0.0016, *G3bp2*: ***p* = 0.0019, *Ptk2:* ***p* = 0.0035, *Ripk1*: ***p* = 0.0072, *Polr1e*: ***p* = 0.0076, *Chuk*: ***p* = 0.0086, *Psmd13*: **p* = 0.0119, *Birc2*: **p* = 0.026, *Traf6*: **p* = 0.0428, *Gtf2i*: **p* = 0.0434, *Csnk2a2*: **p* = 0.045, *Nr2c2*: **p* = 0.047.

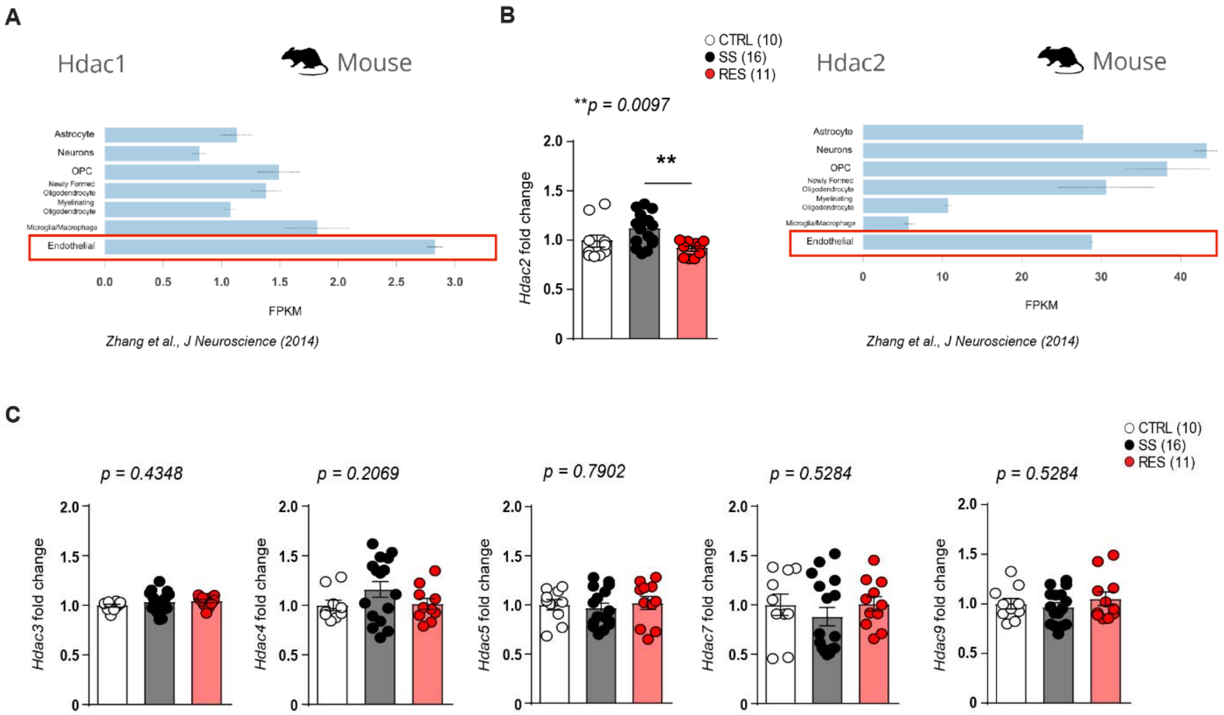

Supp. Fig.10 – Hdacs expression in the NAc following chronic social stress. A) *Hdac1* expression is enriched in endothelial cells compared to other brain cell types. **B**) A significant difference was observed for *Hdac2* in the NAc of SS mice as reported in a previous study (2) (one-way ANOVA: $F_{2,33} = 5.350$; $**p = 0.0097$). However, *Hdac2* expression is higher in neurons and astrocytes vs endothelial cells. **C**) No significant difference was noted for other *Hdacs*.

Supplemental Information - Tables

Table 1 – Human qPCR primers

Gene	Ref Seq #	Assay ID	Forward primer	Reverse primer
CLDN5 (500bp before TS)			5′- CGGGATTTCCGGGCATTCTT-3′	5′- GTCCACACCAGTGGACCTTT-3′
CLDN5 (2700bp before TS)			5′-CTCACTGGTAACGAAGCCCC-3′	5′-AAAGGCAGAAGAGAACCGGG-3′
GAPDH	NM_002046(1)	Hs.PT.39a.22214836	PrimeTime®qPCR primers Exon Location 2-3	
FOXO1	NM_002015(1)	Hs.PT.58.40005627	PrimeTime®qPCR primers Exon Location 1-2	
CTNNB1	NM_001098209 (3)	Hs.PT.58.40551289	PrimeTime®qPCR primers Exon Location 10-11	
HDAC1	NM_004964(1)	Hs.PT.58.38680914	PrimeTime®qPCR primers Exon Location 3-4	
CLDN5	NM_003277(2)	Hs.PT.58.1483777.g	PrimeTime®qPCR primers Exon Location 1-1	

Table 2 – Mouse qPCR primers

Gene	Ref Seq #	Assay ID	Forward primer	Reverse primer
Cldn5	NM_013805(1)		5’-TTTCTTCTATGCGCAGTTGG-3’	5’-GCAGTTTGGTGCCTACTTCA-3’
Gapdh	NM_008084(1)	Mm.PT.39a.1	PrimeTime®qPCR primers Exon Location 2-3	
Cdh5	NM_009868(1)	Mm.PT.58.8747496	PrimeTime®qPCR primers Exon Location 7-8	
FoxO1	NM_019739 (1)	Mm.PT.58.6477586	PrimeTime®qPCR primers Exon Location 1-2	
Ctnnb1	NM_001165902 (2)	Mm.PT.58.12501105	PrimeTime®qPCR primers Exon Location 10-11	
Pecam1 (CD31)	NM_008816(2)	Mm.PT.58.43167370	PrimeTime®qPCR primers Exon Location 7-8	
Ocln	NM_008756(1)	Mm.PT.58.42749240	PrimeTime®qPCR primers Exon Location 7-9	
Mfsd2A	NM_029662(1)	Mm.PT.58.32675283	PrimeTime®qPCR primers Exon Location 13-14	
Nostrin	NM_181547(1)	Mm.PT.58.12259437	PrimeTime®qPCR primers Exon Location 14-16	
Aldh1l1	NM_027406(1)	Mm.PT.58.7775479	PrimeTime®qPCR primers Exon Location 13-15	
Aqp4	NM_009700(1)	Mm.PT.58.9080805	PrimeTime®qPCR primers Exon Location 1-2	
Mbp	NM_001025254 (4)	Mm.PT.58.28532164	PrimeTime®qPCR primers Exon Location 6-9	
Slc17a6	NM_080853(1)	Mm.PT.58.10363705	PrimeTime®qPCR primers Exon Location 9-10	
Hdac1	NM_008228(1)	Mm.PT.58.14183463	PrimeTime®qPCR primers Exon Location 7-8	

Table 3 – Complete demographic for human cohorts

Texas cohort for epigenetic studies

Gender	Age	Post mortem interval	Cause of death
M	31	16	Hypertensive cardiovascular disease
M	63	14	Acute myocardial infarction
M	19	20	Gunshot wound
M	48	15	Mitral valve regurgitation
M	20	21	Blunt force injury
M	60	20	Surgical complication
M	43	15	Hypertensive cardiovascular disease
M	60	11	Hypertensive cardiovascular disease
M	63	12	Acute myocardial infection
M	34	23	Arthersosclerotic cardiovascular disease
M	48	20	Arthersosclerotic cardiovascular disease
M	54	11.4	Hypertensive cardiovascular disease
M	77	13.4	Pancreatic cancer
M	60	27	Acute myocardial infarction

Gender	Age	Post mortem interval	Antidepressant treatment	Cause of death
M	25	21	No	Suicide
M	42	17	No	Suicide
M	24	18	No	Suicide
M	18	22	No	Suicide
M	61	19	No	Suicide

Gender	Age	Post mortem interval	Antidepressant treatment	Cause of death
M	33	18	Yes	Suicide

M	40	18	Yes	Suicide
M	35	9	Yes	Suicide
M	61	20	Yes	Suicide
M	50	23	Yes	Suicide
M	65	14	Yes	Hypertensive cardiovascular disease

Montreal cohort for qPCR analysis and immunostaining

Gender	Age	Post mortem interval	Cause of death	Depressive symptoms	History of abuse	Antidepressant treatment	Alcool	Drugs of abuse
M	47	12	Natural	No	No	No	No	No
M	41	24	Natural	No	No	No	No	No
M	30	30	Accident	No	No	No	No	No
M	19	27.75	Suicide	No	N/A	No	No	No
M	46	19.5	Natural	No	No	No	No	No
M	32	29.5	Accident	No	N/A	No	No	No
M	33	18	Suicide	No	Yes	No	No	No
M	42	63	Accident	No	N/A	No	No	No
M	55	24	Accident	No	Yes	No	No	No
M	46	59	Suicide	No	N/A	No	No	No
M	52	10	Natural	No	N/A	No	No	No
M	29	4	Suicide	No	N/A	No	No	No
M	59	23.5	Accident	No	N/A	No	No	No
M	48	54	Natural	No	No	No	No	No
M	82	46	Accident	No	No	No	No	No
M	21	62	Accident	No	No	No	No	No
M	74	45	Accident	No	No	No	No	No
M	63	48.5	Natural	No	No	No	No	No
F	66	61	Accident	No	N/A	No	Yes	No
F	72	17	Natural	No	No	No	No	No
F	45	44	Suicide	No	Yes	Yes	No	No

F	81	13.5	Natural	No	N/A	No	No	No
F	78	7.5	Accident	No	Yes	No	Yes	No
F	76	7	Accident	No	No	Yes	No	No
F	68	8.5	Natural	No	N/A	No	No	No
M	49	2.5	Suicide	Yes	No	No	No	No
M	53	33.5	Suicide	Yes	Yes	No	No	No
M	38	30	Suicide	Yes	N/A	No	No	No
M	28	36	Suicide	Yes	N/A	No	No	No
M	29	27	Suicide	Yes	No	No	No	No
M	68	43	Suicide	Yes	N/A	No	No	No
M	63	50	Suicide	Yes	N/A	No	No	No
M	48	49	Suicide	Yes	No	No	No	No
M	67	56	Suicide	Yes	N/A	No	No	No
M	52	29	Suicide	Yes	N/A	No	No	No
M	53	14	Suicide	Yes	N/A	No	No	No
M	51	54	Suicide	Yes	N/A	No	No	No
M	39	25.5	Suicide	Yes	N/A	No	No	No
M	49	32	Suicide	Yes	N/A	No	No	No
M	40	22	Suicide	Yes	N/A	No	No	No
M	65	55	Suicide	Yes	No	No	No	No
M	70	85	Suicide	Yes	Yes	No	Yes	No
M	32	51	Suicide	Yes	No	No	No	No
M	71	58	Suicide	Yes	No	No	No	No
F	25	20	Suicide	Yes	N/A	No	No	No
F	55	36	Suicide	Yes	No	No	No	No
F	25	56	Suicide	Yes	N/A	No	No	No
F	32	41	Suicide	Yes	N/A	No	No	No
F	55	2.5	Suicide	Yes	Yes	No	No	No
F	44	60	Suicide	Yes	N/A	No	No	No

M	42	21	Suicide	Yes	Yes	Yes	No	No
M	48	21.5	Suicide	Yes	N/A	Yes	No	No
M	22	24	Suicide	Yes	Yes	Yes	No	No
M	53	41	Suicide	Yes	No	Yes	No	No
M	36	36.5	Suicide	Yes	N/A	Yes	No	No
M	63	24	Suicide	Yes	N/A	Yes	No	No
M	35	31	Suicide	Yes	N/A	Yes	No	No
M	47	2.5	Suicide	Yes	N/A	Yes	No	No
M	65	80	Suicide	Yes	No	Yes	No	No
M	57	59	Suicide	Yes	Yes	Yes	Yes	No
M	64	63	Suicide	Yes	Yes	Yes	Yes	No
M	56	76	Suicide	Yes	No	Yes	No	No
M	47	43	Suicide	Yes	No	Yes	No	No
F	46	15	Suicide	Yes	N/A	Yes	Yes	No
F	40	49.5	Suicide	Yes	N/A	Yes	No	No
F	54	28.5	Suicide	Yes	Yes	Yes	No	No
F	49	14.5	Suicide	Yes	N/A	Yes	No	No
F	36	7.5	Suicide	Yes	No	Yes	No	No
F	55	36	Suicide	Yes	N/A	Yes	No	No
F	59	3	Suicide	Yes	No	Yes	No	No
